# PA28αβ overexpression enhances learning and memory of female mice without inducing 20S proteasome activity

**DOI:** 10.1186/s12868-018-0468-2

**Published:** 2018-11-06

**Authors:** Julia Adelöf, My Andersson, Michelle Porritt, Anne Petersen, Madeleine Zetterberg, John Wiseman, Malin Hernebring

**Affiliations:** 1 Department of Clinical Neuroscience, Institute of Neuroscience and Physiology, Sahlgrenska Academy at the University of Gothenburg, Gothenburg, Sweden; 2 IMED Biotech Unit, Discovery Biology, Discovery Sciences, AstraZeneca, Gothenburg, Sweden; 3 Department of Clinical Sciences, Epilepsy Centre, Lund University, Lund, Sweden

**Keywords:** PA28αβ, Learning and memory, F2 hybrid transgenic mice, Behavioral phenotyping, 20S proteasome, Proteasome capacity, K48-linked protein ubiquitination

## Abstract

**Background:**

The proteasome system plays an important role in synaptic plasticity. Induction and maintenance of long term potentiation is directly dependent on selective targeting of proteins for proteasomal degradation. The 20S proteasome activator PA28αβ activates hydrolysis of small nonubiquitinated peptides and possesses protective functions upon oxidative stress and proteinopathy. The effect of PA28αβ activity on behavior and memory function is, however, not known. We generated a mouse model that overexpresses PA28α (PA28αOE) to understand PA28αβ function during healthy adult homeostasis via assessment of physiological and behavioral profiles, focusing on female mice.

**Results:**

PA28α and PA28β protein levels were markedly increased in all PA28αOE tissues analyzed. PA28αOE displayed reduced depressive-like behavior in the forced swim test and improved memory/learning function assessed by intersession habituation in activity box and shuttle box passive avoidance test, with no significant differences in anxiety or general locomotor activity. Nor were there any differences found when compared to WT for body composition or immuno-profile. The cognitive effects of PA28αOE were female specific, but could not be explained by alterations in estrogen serum levels or hippocampal regulation of estrogen receptor β. Further, there were no differences in hippocampal protein expression of neuronal or synaptic markers between PA28αOE and WT. Biochemical analysis of hippocampal extracts demonstrated that PA28α overexpression did not increase PA28–20S peptidase activity or decrease K48-polyubiquitin levels. Instead, PA28αOE exhibited elevated efficiency in preventing aggregation in the hippocampus.

**Conclusions:**

This study reveals, for the first time, a connection between PA28αβ and neuronal function. We found that PA28α overexpressing female mice displayed reduced depressive-like behavior and enhanced learning and memory. Since the positive effects of PA28α overexpression arose without an activation of 20S proteasome capacity, they are likely independent of PA28αβ’s role as a 20S proteasome activator and instead depend on a recognized chaperone-like function. These findings suggest that proteostasis in synaptic plasticity is more diverse than previously reported, and demonstrates a novel function of PA28αβ in the brain.

**Electronic supplementary material:**

The online version of this article (10.1186/s12868-018-0468-2) contains supplementary material, which is available to authorized users.

## Background

The proteasome is a sophisticated multi-subunit protease comprising the 20S catalytic core and up to two proteasome activators that interact physically with 20S and control substrate entry to its inner proteolytic compartment. PA28αβ is a proteasome activator, which is produced upon interferon-γ stimulation [1] and oxidative stress [2]. Whilst involved in antigen processing and presentation by the major histocompatibility complex I (MHC-I) [3, 4], PA28αβ provides protective functions upon oxidative stress and proteinopathy as demonstrated in both animal and cell model studies [5–9].

Protective effects of PA28αβ have been found in mice, where an overexpression of PA28α specifically in cardiomyocytes lowered the myocardial infarct size upon ischemia/reperfusion (I/R) and preserved ventricular contractility after reperfusion [5]. Cardiomyocyte-specific overexpression of PA28α also prolonged lifespan of a desmin-related cardiomyopathy mouse model while reducing its associated proteinopathy [5]. Cultured rat cardiomyocytes overexpressing PA28α exhibit reduced apoptosis and protein oxidation upon hydrogen peroxide (H_2_O_2_) exposure [6]. Immortalized mouse embryonic fibroblasts (MEFs) exposed to a mild pre-treatment of H_2_O_2_ become less sensitive to a harsh H_2_O_2_ exposure compared to untreated cells. This H_2_O_2_ adaptation requires induction of PA28α [7, 8]. Furthermore, PA28α is essential for protein damage control during mouse ES cell differentiation [9].

The mechanism behind PA28αβ’s protective effects is not known. In vitro studies using purified proteins have shown that PA28αβ can induce degradation of an oxidized protein substrate [7] and exhibit chaperone-like functions in collaboration with Hsp40, Hsp70 and Hsp90 [10].

It is well established that the proteasome system is important for the nervous system to function properly. Proteasome-dependent protein degradation is known to be critical for long-term potentiation (LTP) [11–16], a molecular mechanism central for learning and memory. Proteasome inhibition impairs murine memory and learning analyzed by one-trail inhibitory avoidance [15], taste aversion [16], auditory fear conditioning and context fear conditioning [17]. In addition, the proteasome system regulates synaptic transmission at both presynaptic and postsynaptic terminals in mammalian neurons [18–20]. However, the role of PA28αβ in neuronal function is an almost completely unexplored field. Protein expression patterns in the brain of healthy and disease subjects [21–24] suggest a role in neurodegenerative disease and traumatic brain injury, but no mechanistic studies have been performed.

We have generated a mouse model in which the gene encoding murine PA28α is overexpressed in all analyzed tissues (PA28αOE). The aim of this study is to characterize the behavior and physiology of female healthy adult mice to gain insight into the role of PA28αβ in normal physiological and cognitive functions.

## Results

### Generation and evaluation of the PA28αOE model

**Fig. 1:**
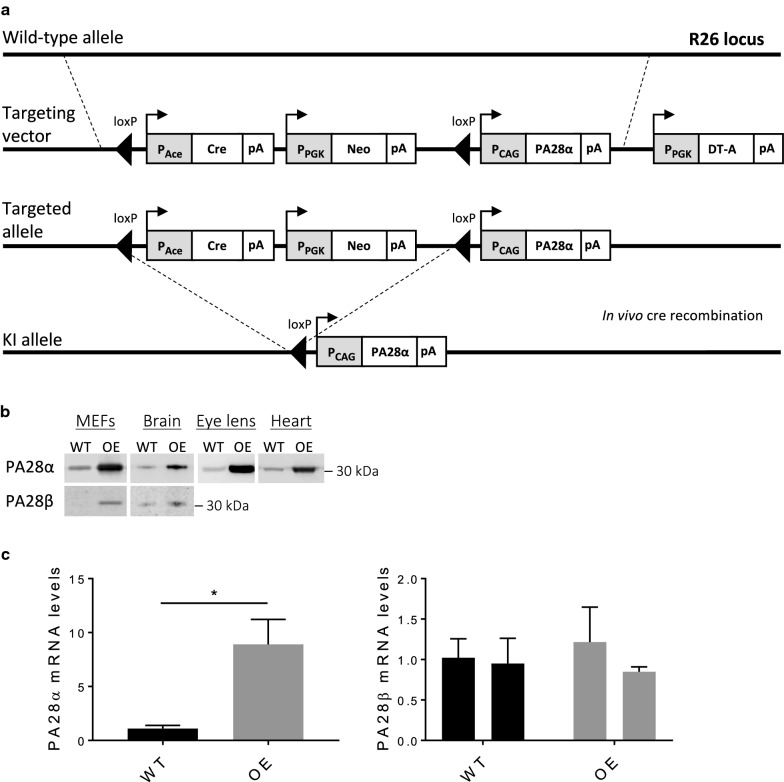
Generation and validation of the PA28αOE mouse model. **a** Structure of the targeting vector, targeted allele and KI allele (PA28αOE). **b** Representative western blots of PA28α in cultivated mouse embryonic fibroblasts (MEFs), frontal cortex and striatum (brain), eye lens and left ventricle of the heart and representative western blots of PA28β in MEFs and frontal cortex and striatum (brain) from litter mates of PA28αOE (OE) and wildtype (WT) C57BL/6 male mice (founder strain). Analysis demonstrated that PA28α is induced fivefold in MEFs (*P *< 1E−5; Student’s *t* test), 3.2-fold in brain (*P *< 0.001), 15-fold in eye lens (*P *= 0.0049), and fourfold in heart (*P *< 1E−6), while PA28β is induced 2.7-fold in brain (*P *= 0.0015) (n = 3). The amount of PA28β was below detection level in WT MEFs, but detected in all OE MEFs (n = 3). **c** Relative levels of mRNA encoding PA28α and PA28β with 36b4 as a reference gene in a mixed sample of frontal cortex and striatum of the brain. Values are mean ± SEM normalized to mean WT-value (*P*_*PA28α*_= 0.028; n = 3). Two different splicing variants of the PA28β transcript were analyzed

A targeting vector for PA28α overexpression (PA28αOE), which included the *CAG* promoter driving the expression of the coding region of *murine PA28*α, was targeted to the murine *Rosa26 locus* (see Fig. 1a). Correct integration in murine embryonic stem (ES) cells and murine splenocytes was confirmed by Targeted Locus Amplification (Cergentis).

Western blot analysis of male PA28αOE C57BL/6 mice (n = 3) confirmed that PA28α was overexpressed in heterozygous PA28αOE eye lens (*P *= 0.0049), left ventricle of the heart (*P *< 1E−6), mouse embryonic fibroblasts (MEFs; *P *< 1E−5) as well as in a mixed sample of frontal cortex and striatum of the brain (*P *< 0.001; Fig. 1b and Additional file 1). PA28α overexpression resulted in an upregulation of PA28β (the other PA28αβ subunit) in MEFs, frontal cortex and striatum from PA28αOE (detected in OE MEFs but below detection level in WT; Fig. 1b and Additional file 1), while PA28β mRNA levels were not affected (Fig. 1c and Additional file 1). This is in line with the previous finding in cardiomyocytes that PA28α overexpression stabilizes PA28β at the protein level [6]. Hence, all components of the PA28αβ complex are present in the tissues analyzed from the PA28αOE mouse model.

### Female C57BL/6N × BALB/c F2 hybrid mice

**Fig. 2:**
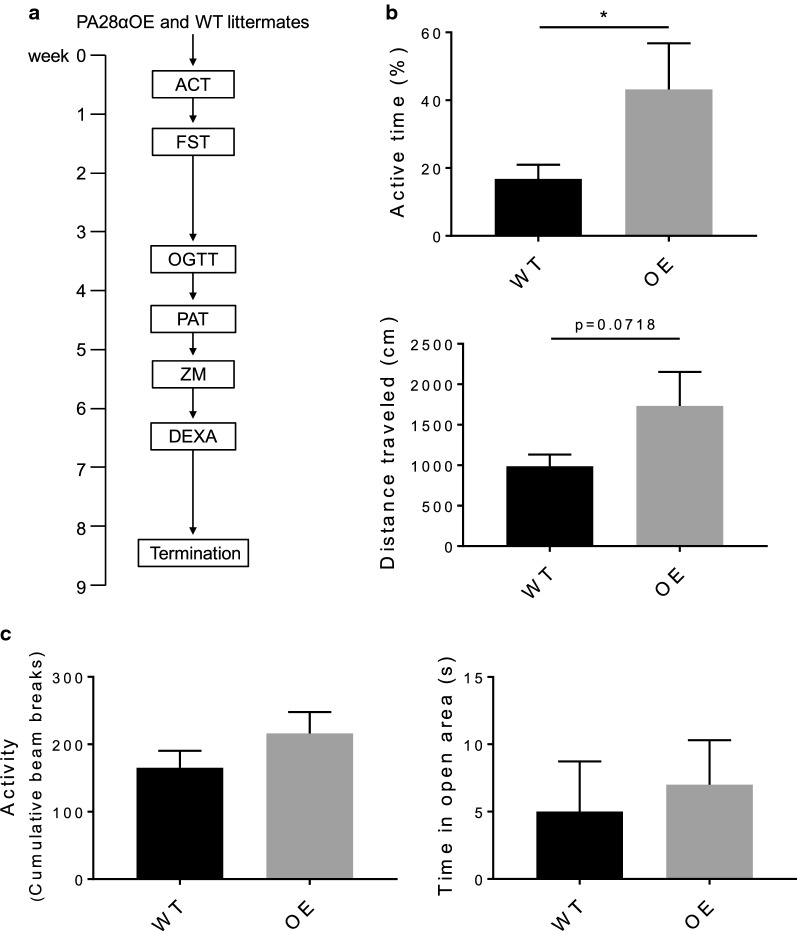
PA28αOE mice exhibit reduced depressive-like behavior. **a** Order of exposure to behavioral and physiological analysis. Female PA28αOE and WT F2 hybrid litter mates at the age of 6–7 months were subjected to the following analysis: activity box (ACT), forced swim test (FST), oral glucose tolerance test (OGTT), passive avoidance test using the shuttle box system (PAT), zeromaze anxiety test (ZM), and dual energy X-ray absorptiometry (DEXA); n_PA28αOE_ = 6 and n_WT_ = 9–10 (one WT mouse was injured during PAT and needed to be removed, thus n_WT_ = 10 until PAT and n_WT_ = 9 thereafter). **b** Forced swim test is considered a measurement of depressive-like behavior, in which low active time and passivity are signs of depression. PA28αOE female mice demonstrate increased active time (*P *= 0.0476; Student’s *t* test) and distance travelled (*P *= 0.0718) compared to WT littermates. Values are mean ± SEM. **c** Analysis of PA28αOE and WT mice using the zeromaze system to study anxiety-related behavior. A mouse is placed at the entrance of a closed quadrant and monitored for 5 min as regards of their activity, latency to enter open arm and time spent in open and closed arm. Graphs show activity in closed area and time spent in open area for both days, which are considered the most relevant measures of anxiety-like behavior. Values are mean ± SEM; n_PA28αOE_ = 6 and n_WT_ = 10

Crosses between two inbred strains, hybrids, tend to be more genetically vigorous and less sensitive to adverse environmental conditions than inbred strains. To take advantage of these characteristics we generated C57BL/6N × BALB/c F2 hybrids for phenotypic profiling of the PA28αOE mouse model. We chose to analyze the mice at the age of 7–8 months, approximately corresponding to a human age of 32–34 years [25], to ensure focus on adult homeostasis. 10 wildtype (WT) and 6 PA28αOE heterozygous female F2 hybrid littermates were subjected to physiological and behavioral phenotypic profiling as outlined in Fig. 2a.

### PA28αOE mice displayed no differences in body composition or immunological profile

PA28αOE mice appeared healthy and energetic, and were visually indistinguishable from WT with respect to appearance and behavior. There were no statistically significant differences observed in the physiological parameters body temperature, body weight and length, fat mass, lean mass and bone density (Additional file 2). PA28αOE and WT mice had similar immunological profiles with no differences in the levels of circulating granulocytes, monocytes, lymphocytes, NK cells, B cells, T cells, CD4 + T cells or CD8 + T cells (assayed at termination, Additional file 3), and displayed similar response in oral glucose tolerance test (OGTT, Additional file 4).

### PA28αOE mice show decreased depressive-like behavior and increased learning

**Fig. 3:**
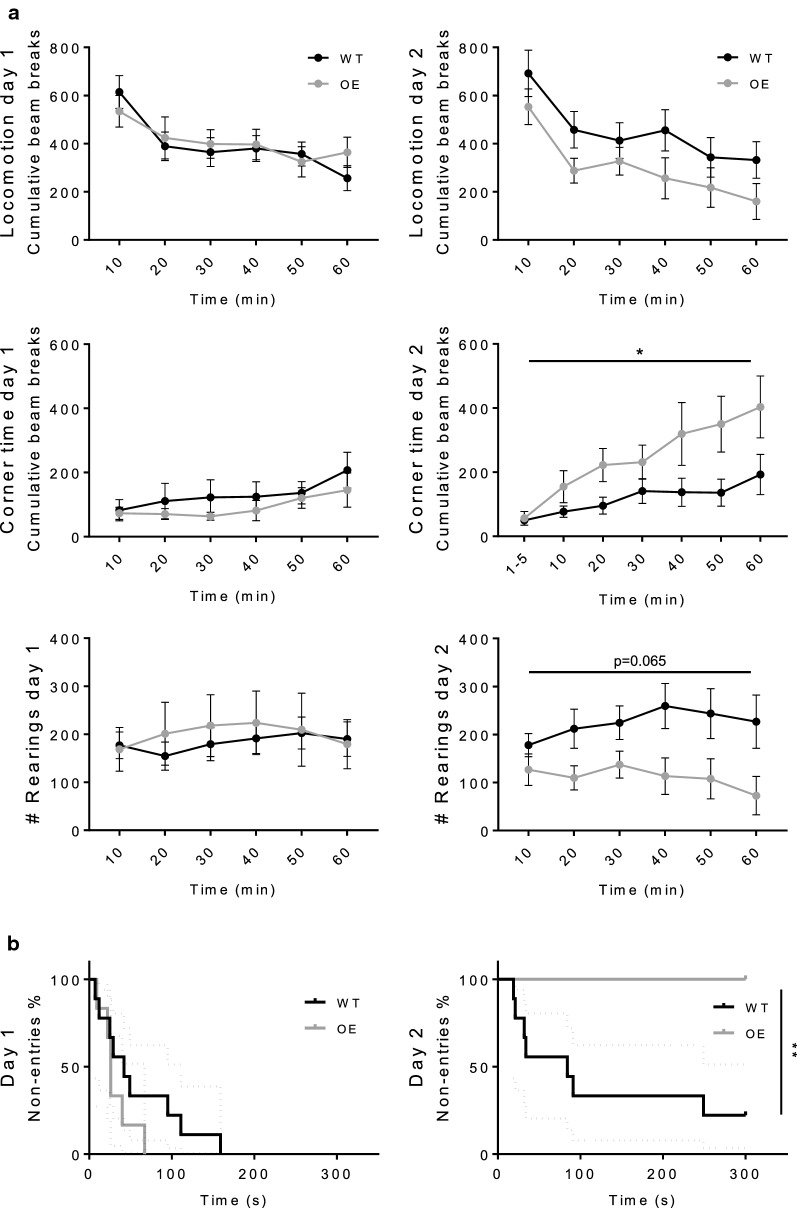
PA28αOE mice exhibit improved learning/memory. **a** Activity box measurements of exploratory behavior; locomotion, rearing and corner time in novel (day 1) and acquainted (day 2) environment. On day 2, PA28αOE females spent longer time in the corners (*P *= 0.034; two-way ANOVA repeated measurements, followed by Sidak test, F(1, 14) = 5498), and exhibited a strong tendency of reduced rearing [*P *= 0.065; F(1, 14) = 3992], both of which are indicators of more proficient habituation. Locomotion on day 2 between PA28αOE and WT is not statistically different (*P *= 0.19). Values are mean ± SEM. **b** Shuttle box passive avoidance test assesses learning and memory capability. On day 1 (*P*_*day1*_= 0.194, Mantel–Cox survival test) a small electric shock was given to PA28αOE female mice and their wildtype female littermates upon voluntarily entering a dark compartment. As shown, on day 2 there is a significant difference between PA28αOE and WT in re-entering the compartment (*P*_*day2*_= 0.0056). Maximum assay time was 300 s (i.e. no entry = 300 s). Dashed lines correspond to 95% confidence interval

In the behavioral examinations, PA28αOE mice displayed a 400% increase in mean active time in the forced swim test (FST, Fig. 2b and Additional file 5; *P *= 0.047), indicating a reduction in depressive-like behavior compared to WT. This was not explained by a change between groups in terms of general anxiety or physical activity, as there were neither differences in anxiety levels, measured in the elevated zeromaze (Fig. 2c and Additional file 5), nor general physical activity as determined by activity box measurements (Locomotion, Fig. 3a and Additional file 6).

To determine if the decreased depressive like behavior displayed by the PA28αOE mice resulted in improved cognitive functions, we compared the time the animals spent in corners and the number of rearings (standing on hind legs, an indication of vigilance) during the first day of the activity box experiment (new environment) to the second day of trail (acquainted environment). On day 2, PA28αOE spent significantly more time in the corner (Fig. 3a and Additional file 6; *P *= 0.034) and exhibited a strong inclination to reduced rearing (*P *= 0.065), indicating enhanced intersessional habituation of PA28αOE compared to WT [26, 27]. A difference in learning capacity was apparent when the animals were exposed to the shuttle box passive avoidance test, all PA28αOE mice (6/6) stayed out of the dark compartment on day 2, where they experienced a mild electric shock the previous day, while only 2 out of 10 WT did the same (Fig. 3b and Additional file 6; *P *= 0.0056). This behavior did not result from a change in pain tolerance between WT and PA28αOE, determined by direct response in the shuttle box assay (see Methods section) and by the tail-flick method (Additional file 7). Taken together, PA28αOE mice display decreased depressive-like behavior and an increased capacity for learning compared to their WT littermates.

### The cognitive effects of PA28αOE are female specific

As the previous experiments were performed with female mice, we wanted to determine whether these behavioral effects of PA28αOE were gender specific. To this end, we subjected male C57BL/6N × BALB/c F2 hybrids (litter mates to the females analyzed) to the same cognitive behavioral tests. Male PA28αOE mice did not, however, display an increase in activity compared to WT when subjected to the forced swim test (n ≥ 5; Additional file 8). Neither did they show any significant changes in learning compared to WT in the shuttle box passive avoidance, nor were there any indications of enhanced habituation in the male PA28αOE mice compared to WT in activity box measurements (n ≥ 5; Additional file 8). Thus, we could not find any effect of PA28α overexpression in male mice on cognitive behavior in our behavioral tests suggesting that the effects of PA28αOE overexpression are female specific.

### PA28αOE mice display no change in serum estrogen or hippocampal estrogen receptor regulation

As the declarative component of passive avoidance memory formation is formed in the hippocampus and fluctuating levels of estrogen directly affect hippocampal memory function [28], differences in estrogen levels between animal groups could explain the results obtained in our behavioral assays. To determine if this was the case, blood serum levels of estrogen was determined by ELISA, showing no differences in estrogen levels between PA28αOE and WT (Additional file 9), nor in the levels of S105-phosphorylated estrogen receptor β from hippocampal extracts (Additional file 9). This suggests that the resulting differences in behavior are neither a result of differences in serum levels of estrogen nor hippocampal regulation of estrogen receptor β.

### PA28αOE mice exhibit no differences in hippocampal protein expression of neuronal or synaptic markers

As proteasome-dependent protein degradation is crucial for long-term potentiation (LTP) [11–16] we investigated if there were global changes in the amount of AMPA receptors and/or synaptic markers in the PA28αOE mice that could explain their increased learning capacity. Isolation of hippocampus (9 WT and 6 PA28αOE female F2 hybrids) and subsequent WB analysis revealed no differences in neuronal density of neurons, measured by expression of the neuronal nuclear marker NeuN (Additional file 9) or synaptic density, measured as expression of adhesion molecule N-Cadherin, together with the presynaptic vesicle glycoprotein Synaptophysin and the excitatory postsynaptic density protein PSD-95 (Additional file 9). The AMPA receptor subunits GluA1 and GluA2 did not differ in expression between animal groups, nor the expression of Spinophilin, a postsynaptic density protein involved in spine formation (Additional file 9). However, expression of GluA2, Synaptophysin and GluA1 varied greatly but correlated to each other and to the level of S105-phosphorylated estrogen receptor β (Additional file 9; correlation coefficients: CC_GluA2-Synphys_: 0.93; CC_GluA2-GluA1_ = 0.92; CC_GluA2-S105-ERβ_ = 0.72; CC_Synphys-GluA1_ = 0.83; CC_Synphys-S105-ERβ_ = 0.61; CC_GluA1-S105-ERβ_ = 0.84).

**Fig. 4:**
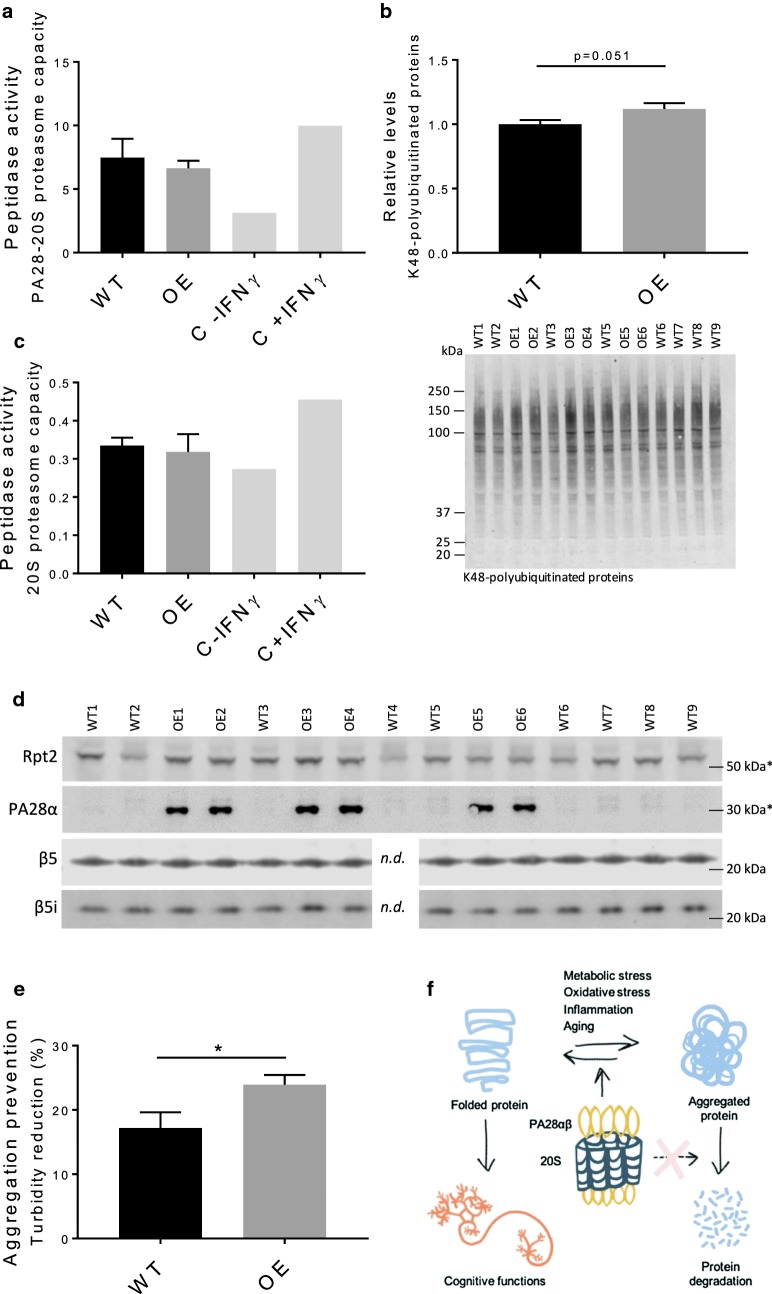
PA28α overexpression does not increase hippocampal PA28-dependent proteasome activity, but enhances aggregation prevention capacity in the hippocampus. **a** The PA28-dependent proteasome capacity determined by suc-LLVY-AMC digestion (i.e. β5/β5i chymotrypsin-like activity) under PA28–20S optimizing conditions, with interferon-γ treated MEFs serving as positive control (compare C – FNγ to C + IFNγ). Activity presented is activity inhibited by the proteasome-specific inhibitor epoxomicin (5 µM), which corresponded to 70–98% of total activity (see Methods). Values are mean ± SEM; n_PA28αOE_ = 3 and n_WT_ = 4; differences are not statistically significant (*P *= 0.72). **b** Representative blot of K48-linked polyubiquitin western analysis and quantification of K48-linked polyubiquitinated protein signal from western analysis, *P *= 0.051; Student’s *t* test). Values are mean ± SEM; n_PA28αOE_ = 6 and n_WT_ = 8. **c** 20S proteasome capacity (in the presence of 0.02% SDS) in protein extracts made from PA28αOE and WT right hippocampus, values are mean ± SEM; n_PA28αOE_ = 3 and n_WT_ = 4. **d** Western analysis of the proteasome related markers Rpt2 (19S subunit), β5 (20S) and β5i (20Si) in protein extracts made from PA28αOE and WT left hippocampus. PA28α is induced 13-fold in PA28αOE hippocampus (*P *< 1E−12; Student’s *t* test). *Estimated kDa marker placement based on 20 kDa and 37 kDa marker bands. “n.d.” = not included in assay due to limited amount of extract. **e** Aggregation prevention of heat-sensitive luciferase in the presence of hippocampal protein extracts at 42 °C. Luciferase aggregation prevention capacity was calculated as percentage of non-aggregated luciferase compared to samples without cell extracts. Boiled extracts served as negative control and did not prevent aggregation. Values are mean ± SEM; n_PA28αOE_ = 9 and n_WT_ = 10 (*P *= 0.036; Student’s *t* test). **f** A model of PA28αβ effects on cognitive functions through its role as a chaperone rather than a 20S proteasome activator

LTP in the hippocampus is dependent on activation of calcium calmodulin kinase II [29] and is positively modulated by cAMP response element-binding protein [30] suggesting that increased activation of these could correlate to enhanced learning. We could, however, find no differences in phosphorylated CaMKII or phosphorylated CREB between PA28αOE and WT hippocampi (Additional file 9).

Hence, global expression levels of neuronal, synaptic or LTP markers cannot explain the cognitive differences observed between WT and PA28αOE female mice.

### PA28αOE mice do not demonstrate induced proteasome activity

The prevailing view of PA28αβ´s molecular function in the cell is activation of the 20S proteasome. We performed proteasome activity assays on hippocampal extracts from WT and PA28αOE female mice to investigate whether the cognitive effects observed upon PA28α overexpression are associated with such activation. The interactions between the 20S proteasome and its different regulators are perturbed or compromised by mutually exclusive extraction conditions [31–33]. Thus, to detect PA28-dependent activation of 20S proteasome capacity (digestion of fluorogenic peptides in vitro), conditions specifically optimizing the interaction between 20S and PA28αβ need to be used [e.g. no salt; 31, 32].

We detected a threefold increase in PA28-dependent proteasome capacity upon IFN-γ treatment after using conditions optimizing PA28–20S interaction (MEFs, positive control, Fig. 4a and Additional file 10). However, in PA28αOE hippocampi there was no induction in PA28-dependent proteasome capacity compared to WT hippocampi (Fig. 4a and Additional file 10; n ≥ 3, animals used in the phenotypic profiling). This was unexpected since PA28α is capable of self-assembly in vitro into a heptamer ring [34, 35], which can activate 20S proteasome capacity [36]. To further analyze this, PA28-dependent proteasome capacities of PA28αOE and WT MEFs were determined, also demonstrating a lack of induction in PA28αOE (Additional file 11, 12).

Western analysis of hippocampal K48-linked polyubiquitinated proteins, generally targeted for proteasomal degradation, showed a clear trend of increased levels in PA28αOE (Fig. 4b and Additional file 13; *P *= 0.051), indicating reduced 26S proteasome activity and in line with PA28α overexpression being unable to activate proteasome activity. 20S proteasome capacity was not induced in PA28αOE hippocampi (Fig. 4c and Additional file 10) and hippocampal levels of proteasome related markers Rpt2 (19S subunit), β5 (20S) and β5i (20Si) did not differ between WT and PA28αOE (Fig. 4d and Additional file 13), while PA28α overexpression in PA28αOE hippocampi was verified (Fig. 4d).

For these reasons, we conclude that the improved cognitive functions observed upon PA28α overexpression arise without an increase in 20S proteasome activity, and are therefore likely not dependent on this molecular function of PA28αβ.

### PA28αOE hippocampus extracts prevent aggregation more efficiently than WT

PA28αβ has a recognized chaperone-like activity not necessarily coupled to its role as a 20S proteasome activator [10, 37]. Therefore, WT and PA28αOE female hippocampal extracts were analyzed to determine their capacity to prevent aggregation of heat sensitive luciferase. Hippocampal extracts from PA28αOE were more efficient in preventing aggregation than WT hippocampal extracts (Fig. 4e and Additional file 13; *P *= 0.036). Thus, as depicted in Fig. 4f, chaperone-like functions of PA28αβ, rather than its role as a proteasome activator, are likely part of the mechanism behind the observed cognitive effects in PA28αOE.

## Discussion

This study couples, for the first time, PA28αβ to neuronal function and demonstrates that PA28α overexpression reduces depressive-like behavior and enhances learning/memory in female mice without inducing 20S proteasome activity. Instead, our data suggest that the observed effects on cognitive capacity is exerted by PA28αβ chaperone-like functions.

The forced swim test is an established behavioral model to assess emotional state of rodents [38]. The increased activity time of PA28αOE mice compared to WT could point to an antidepressant action of overexpressing PA28α, though a direct effect of PA28α on enhancing locomotion activity could not be ruled out. We found no difference, however, between WT and PA28αOE mice in general locomotor activity, assessed in the activity box, supporting that the effect observed in the forced swim test is a stress coping mechanism of PA28αOE mice.

Open-field tests are traditionally known to assess emotionality and anxiety-like behavior and are also acknowledged to assay locomotion and exploratory behavior [39–41]. In a new environment, the innate behavior of mice is to explore, specifically the periphery, and avoid the open areas of the box. Habituation is considered the simplest form of learning and memory [26] and can be measured intrasessionally (within-session) which is proposed to primarily reflect adaptation [42] or intersessionally (between sessions) by repeated exposures, reflecting learning and memory [27].

We found no differences in the behavior between PA28αOE and WT during day 1 in the activity box. This indicates that there is no difference between WT and PA28αOE mice in general locomotor activity, adaptation (intrasessional habituation) or anxiety-like behavior; the latter confirmed by zeromaze anxiety analysis. However, on day 2, PA28αOE mice changed their behavior and spent more time in the corner and tended to reduce their number of rearings. Increased corner time and reduced rearing are both indicators of anti-exploratory behavior signifying that PA28αOE mice habituate faster. The increased capacity for learning was further confirmed in the shuttle box passive avoidance test. On day 2, no PA28αOE female mice entered the avoidance-trained area in the shuttle box, in stark contrast to WT littermates. Thus, results from both activity box analysis and shuttle box passive avoidance test demonstrate an increased learning capacity of PA28αOE female mice.

Remarkably, none of the cognitive behavioral effects from PA28α overexpression were observed in male littermates, indicating that these effects are female specific. Results in the forced-swim test have been shown to be dependent on several factors, such as age of animals, strain and gender [38, 43]. A possible explanation for our results could be an innate difference between male and female mice in depressive-like behavior that is reversed by PA28α overexpression, which in females unleashes positive effects on cognitive functions. Comparing male and female performance in the forced swim test shows a trend for a higher baseline activity in males, though not statistically significant. As depressive-like behavior and function of the hippocampus are closely linked [44], the increased capacity for learning could point to a direct effect of PA28αβ in the hippocampus. Upon examination, we found no alterations in serum estrogen levels or hippocampal regulation of estrogen receptor β that could shed light on the mechanism behind the sex difference. Further, no key neuronal or synaptic markers differed in expression between PA28αOE and WT hippocampi.

Proteasome-dependent protein degradation is well known to play central roles in long term potentiation, regulation of synaptic transmission, and synaptic plasticity. However, this study presents data strongly indicating that the effects of PA28α overexpression on memory/learning are not dependent on PA28αβ as a proteasome activator. Analysis of hippocampi from WT and PA28αOE littermates revealed that PA28α overexpression did not increase PA28-dependent 20S peptidase activity or decrease K48-linked polyubiquitin levels. It is important to note that, even though data presented here demonstrates that proteolysis is not generally enhanced in PA28αOE, a change in individual peptides/proteins, although unlikely, cannot be excluded. In addition, an increase in neuronal proteasome activity has been observed in response to learning [15, 45] and it is possible that enhanced PA28αβ expression specifically modulates this complex neuronal activation state in a manner leading to improved memory, while baseline proteasome activity is unaltered.

Previous studies overexpressing PA28α have demonstrated a minor increase in proteasome activity [6, 46]. However, none of those experiments included a positive control (interferon-γ treated cells or purified 20S ± PA28αβ) and were either not conducted under PA28–20S optimizing conditions [6] or not negatively controlled [specific 20S proteasome inhibitor; 46]. In the in vivo study of cardiomyocyte PA28α overexpression [5], enhanced proteasome function was verified by expression of the reporter gene GFPdgn (GFP fused to degron CL1) [5, 47]. Degradation of GFPdgn is however not only dependent on the proteasome itself, but is also a reflection of the efficiency in recognition and unfolding of this particular substrate. The reduction in GFPdgn observed upon PA28α overexpression may thus instead be a result of PA28αβ chaperone functions.

As opposed to proteasome activation, our data suggest that the observed effects on cognition is exerted by PA28αβ chaperone-like functions, since hippocampal extracts from PA28αOE prevented protein aggregation more efficiently than hippocampal extracts from WT. PA28αβ has previously been shown to be able to collaborate with Hsp40, Hsp70 and Hsp90 to refold a denatured protein substrate [10]. In addition, PA28αβ can compensate for Hsp90 functions in major histocompatibility complex class I antigen processing [37].

As a chaperone, PA28αβ may play a direct role in neuron protein homeostasis and metabolism, affecting firing, signaling, action potential generation as well as vesicular transport and release. Improved learning in PA28αOE mice could be due to an elevated capacity for cellular memory formation, i.e. an enhanced probability for inducing LTP or an increase in the actual magnitude of LTP. Induction and expression of LTP is ultimately expressed as number of AMPA-receptors in the synapse [48] but whole-hippocampi analysis of GluA1 and A2 did not reveal any differences between WT and PA28αOE. A chaperone-like activity could be an explanation for this, as PA28αβ could exert its effect by increasing AMPA-receptor stability, rather than number, thus increasing availability of AMPA-receptors in the membrane and subsequently the likelihood of AMPA-recruitment to the synapse.

In addition, or alternatively, to a direct role in neuron function, PA28αβ chaperone-like activity could be part of the mechanism behind its protective effects against oxidative stress and proteinopathy, and thus the positive effects on cognitive capacity by PA28α overexpression could be a result of neuroprotection. Oxidative stress and proteinopathy are central for the progression and/or detrimental effects of many diseases that cause decline in cognitive functions, including neurodegenerative diseases and neuronal injury after stroke and head trauma [49, 50]. Hence, PA28α overexpression may increase the fitness and exert effects protecting against these diseases, perhaps acting at the core of early aging events.

## Conclusions

In this study, we demonstrate that overexpression of PA28α and concomitant upregulation of PA28β protein reduces depressive-like behavior and enhances learning and memory in female mice. The overexpression of PA28α does not increase PA28αβ-dependent proteasome activity but could still be linked to its protective functions upon oxidative stress and proteinopathy. The underlying mechanism to these protective effects may instead involve chaperone-like functions of PA28αβ.

## Methods

### Animal care, diets and termination

C57BL/6N (Charles River, Lyon, France), BALB/c (Harlan Laboratories, Horst, the Netherlands) and C57BL/6N × BALB/c F2 hybrid mice were housed in a temperature controlled room (21 °C) with a 12:12 h light–dark cycle (dawn: 5.30–6.00 am, dusk: 5.30–6 pm) and controlled humidity (45–55%). They were checked daily had free access to water and regular chow diet (R3; Lactamin, Kimstad Sweden) containing 12% fat, 62% carbohydrates, and 26% protein (energy percentage), with a total energy content of 3 kcal/g. At termination, mice were euthanized under 5% isoflurane anesthesia and decapitated. Blood samples for hematology were collected by intra-cardiac puncture, tissues were isolated, directly transferred to dry ice, and kept at − 80 °C until biochemical analyses.

### Generation of *PA28*α*OE* mice

A knock-in strategy was used to target the murine *Rosa26* locus in order to generate mice carrying a murine PA28α overexpression cassette at this site. The targeting vector was built using homologous recombination in bacteria [51] and a C57 mouse BAC served as template for the extraction of *Rosa26* homology arms. The targeting vector contained the *CAG* promoter [52] driving the expression of the coding region of *murine PA28*α and a rabbit β-globin poly (A) signal (CAG-PA28α-pA) and a neomycin phosphotransferase (Neo) selectable marker cassette. The *PGK*-*gb2*-*neo* cassette with CAG-PA28α-pA was inserted into a *Rosa26* targeting vector comprised of a 1.5 kb 5′ and 5 kb 3′ homology arms of *Rosa26*, and a PGK-diphtheria toxin A (*DTA*) gene for negative selection (Fig. 1a). The Neo selectable marker cassette, which was flanked by *loxp* sites, was deleted in the germline of the chimeric mice generating the KI allele using a self-excising Neo strategy. After linearization, the targeting construct was electroporated into C57BL/6N mouse embryonic stem (ES) cells which were then grown in media containing G418 (200 μg/ml). Thus, the PA28αOE mouse line was established on a pure C57BL/6 genetic background. PCR screens and Targeted Locus Amplification (Cergentis, Utrecht, the Netherlands) analyses revealed clones that had undergone the desired homologous recombination event. Several of these clones were expanded and injected into Balb/cOlaHsd blastocysts to generate chimeric males which were then bred to C57BL/6JOlaHsd females and black-coated offspring were genotyped on both sides of the homology arms for correct integration into the *Rosa26* locus.

### SDS–PAGE and Western blot analysis

MEFs, brain sample containing frontal cortex and striatum, left ventricle of the heart, and hippocampi were lysed with a modified RIPA buffer (50 mM Na_2_HPO_4_ pH 7.8; 150 mM NaCl; 1% Nonidet P-40; 0.5% deoxycholate; 0.1% SDS; 1 mM DTPA; 1 mM pefablock). Cell debris was removed by centrifugation at 5000 g for 10 min and protein concentration was determined using the Pierce™ BCA Protein Assay Kit (Thermo Fisher Scientific). Eye lenses were lysed by sonication (Branson Ultrasonic Corp., Danbury, CT, USA) in PBS [53]. Samples were prepared for SDS–PAGE as described [54], separated by sodium dodecyl sulfate (SDS)-polyacrylamide gel electrophoresis (PAGE), transferred onto a nitrocellulose membrane (Invitrogen, Bleiswjik, the Netherlands) and probed with rabbit mAb PA28α (#9643; Cell Signaling Technology, Inc., Leiden, the Netherlands), rabbit pAb PA28β (#2409), rabbit mAb GluA2 (#13607), rabbit pAb N-Cadherin (#4061), rabbit pAb GluA1 (#13185), rabbit pAb β5/PSMB5 (#11903), rabbit mAb Phospho-CaMKII Thr286 (#12716), rabbit mAb Phospho-CREB Ser133 (#9198), rabbit pAb polyubiquitin K48-linkage specific (ab190061; Abcam, Cambridge UK), goat pAb PSD-95 (ab12093), rabbit pAb GluA1 (ab31232), rabbit mAb Synaptophysin (ab32127), rabbit pAb Spinophilin (ab203275), rabbit mAb NeuN (ab177487), rabbit pAb Estrogen Receptor beta phospho S105 (ab62257), rabbit pAb Rpt2/S4 (ab3317), or rabbit pAb β5i/LMP7 (ab3329). IRDye 800CW-labelled goat anti-rabbit, 680CW-labelled goat anti-mouse, 800CW-labelled donkey anti-goat IgG antibodies (LI-COR Biosciences, Cambridge, UK) were used for detection and blots were analyzed with the Odyssey infrared imaging system and software (LI-COR Biosciences), except for lens samples for which HRP-conjugated secondary Ab was used and luminescence after ECL reaction was imaged using ImageQuant LAS 500 (GE Healthcare, Piscataway, NJ, USA). Blots were quantified using the ImageJ software. Equal total protein present of each sample on the membrane was confirmed using the Novex reversible membrane protein stain (IB7710, Invitrogen) according to manufacturer’s instructions.

### RNA extraction and quantitative (qPCR) analysis

Total RNA was extracted using Stat60 (CS-502, Tel-Test Inc) as per manufacturer’s recommendations. cDNA was synthesized on 1 µg total RNA using the High-Capacity cDNA Reverse Transcription Kit (#4368814 Applied Biosystems, Thermo Fisher Scientific) according to manufacturer’s instructions. Synthesized cDNA was analyzed in triplicates by qPCR using iQTM SYBRH Green Supermix and the QuantStudio 7 Flex system (Applied Biosystems, Thermo Fisher Scientific). For primer sequences, see Additional file 14.

### Study design of physiological and behavioral phenotypic profiling

10 Wildtype (WT) and 6 PA28αOE heterozygous female F2 hybrid littermates at the age of 6 months were subjected to a 2-month protocol of physiological and behavioral phenotypic profiling as outlined in Fig. 2a. The mice were housed 4 in each cage with 2 WT-cages, 1 PA28αOE-cage, and 1 mixed cage. The genotype was not indicated on the cage, and the animal number to genotype was not decoded until after data analysis. The animals were analyzed cage by cage in the following order: WT-cage 1, PA28αOE-cage, WT-cage 2, mixed cage. Shuttle box and zeromaze were performed in 3 rounds with 6 mice each round as follows: (1) 4 WT from WT-cage 1 and 2 PA28αOE from PA28αOE-cage, (2) 2 PA28αOE from PA28αOE-cage and 2 WT from WT-cage 2, (3) 2 WT from WT-cage 2 and 2 PA28αOE and 2 WT from mixed cage. Activity box was performed in 2 rounds with 8 mice each round as follows: (1) 4 WT from WT-cage 1 and 4 PA28αOE from PA28αOE-cage, (2) 4 WT from WT-cage 2 and 2 WT from WT-cage 2 and 2 PA28αOE and 2 WT from mixed cage. All animal experiments were carried out at 10–11 am, except activity box that was carried out at 10–12 am.

### Activity box

Activity box is an open field activity-like test to study general activity, exploratory behavior, signs of anxiety, stress and depression [55]. The mice are three dimensionally recorded by infrared sensors built into the walls (8Lx8Bx8H) of a sound-proof opaque box (50 × 50 × 50 cm) with a low intensity lamp into the lid of the box (Kungsbacka mät och regler, Fjärrås, Sweden). The mice were placed in the middle of the box and recorded for 1 h in this novel environment. On the following day, they were recorded again in the—now considered—acquainted environment. The parameters recorded as events/5 min were horizontal activity, peripheral activity, rearing activity, peripheral rearing, rearing time, locomotion, and corner time.

### Forced swim test

This test is performed to analyze mice for signs of depression [55, 56]. The assembly consists of a transparent plexiglas cylinder with 25 cm inner diameter and 60 cm in length with a grey, circular plastic platform hanging from wires on the outside of the cylinder, approximately 20 cm from the top (bespoke construction, AstraZeneca Gothenburg), and filled with room tempered (22 °C) water in level with the platform. A single mouse is placed on the water surface inside the cylinder and its behavior is monitored by a video camera placed directly above the cylinder for 6 min and 20 s, of which the last 4 min are used in calculation (MouseTracker analysis software).

### Oral glucose tolerance test (OGTT)

Oral glucose tolerance test (OGTT) baseline measurements were obtained after 5 h of fasting, followed by oral glucose dosing (6.7 ml/kg). Insulin levels was measured with Ultra-sensitive mouse insulin ELISA kit (Crystal Chem, Zaandam, Netherlands) according to manufacturer’s instructions and glucose levels by AccuChek mobile blood glucose meter (Roche Diagnostics Scandinavia, Solna, Sweden) at baseline and after 15, 30, 60, 120 min from dosing.

### Shuttle box passive avoidance test

Passive avoidance testing was performed using the shuttle box system (Accuscan Instruments Inc., Columbus, OH, USA). This test is used to study memory performance in mice and is carried out over 2 days [55, 57, 58]. The system consists of a cage centrally divided by a wall into two compartments, one of which has transparent walls (the bright compartment) while the other is covered from all sides with opaque walls (the dark compartment). Both chambers are equipped with sensors that determine the location of the mouse and the central wall has a mechanical sliding door that can be programmed to open or close. The cage floor is made of stainless steel grid, which can deliver a mild electric shock to the mouse upon certain stimuli. On the first day, a mouse is released into the well-lit compartment and tends to migrate to the dark compartment when the central door opens (30 s after mouse entry). Upon entry to the dark compartment, the central door closes and the mouse is exposed to a mild electric shock (0.3 mA). Intensity of pain response was monitored. All mice responded to the electric shock by a vocal response (“beep”) and a jump, indicating similar strength of discomfort. On the second day, the mouse is released as before into the well-lit compartment and when the central door opens, may or may not enter the dark compartment. The time taken to enter the dark compartment is recorded on both days and a longer interval or no entry on the second day indicates memory response. Maximum assay time is 300 s each day.

### Elevated zeromaze monitoring system

The  elevated zeromaze system (Accuscan Instruments Inc.) was used to study anxiety-related behavior [55, 59, 60]. The maze is made up of a circular Perspex platform, elevated 75 cm above the floor, 5 cm wide and 40 cm inner diameter, equally divided into four quadrants, of which two quadrants on opposite sides of the platform are closed by 30 cm high Perspex transparent walls with photocell transceivers, while the other two quadrants are open and bordered by a Perspex lip (0.5 cm high), a security and tactile guide on the open quadrants. During testing, a mouse is placed at the entrance of a closed quadrant and monitored for 5 min. Activity in closed arm, latency to enter open arm, and time spent in open and closed arm are the parameters analyzed.

### Body composition and core temperature

Core body temperature of the mice was obtained with a rectal probe thermometer (ELFA AB, Sweden). Under 2% isoflurane sedation the mice were analyzed by dual energy X-ray absorptiometry (DEXA) using Lunar PIXImus Densitometer (GE Medical Systems, Madison, WI, USA) to determine body fat (g), body fat (%), lean body mass (g), and total BMD (g/cm^2^) [55].

### Immunoprofiling of peripheral blood

Blood samples for hematology (in EDTA tubes, Microvette CB300, Sarstedt, Nürnbrecht, Germany) were collected from the left atrium of the heart under isoflurane anesthesia, prior to necropsy. Leucocytes and erythrocytes were isolated by centrifugation and stained with 1:50 dilutions of MS CD45 HRZN V500 mAb (#561487; BD Diagnostics, Stockholm, Sweden), MS F4/80 PE T45-2342 (#565410), MS CD4 PERCP mAb (#561090), MS CD19 APC mAb (#561738), CD8 APC-Cy7 mAb (#561967), NK1.1 FITC mAb (#553164), and CD3e conjugated to BD Horizon V450 (#560804). Erythrocytes were lysed with BD FACS lysis buffer and analyzed using flow cytometry (FACS Fortessa, BD Bioscience, Stockholm, Sweden) with appropriate filter settings, gating on live cells.

### Blood serum preparation and β-estradiol detection

Blood samples for blood serum preparation were collected from the left atrium of the heart under isoflurane anesthesia, prior to necropsy, incubated at room temperature for 30–45 min, and coagulates were removed by centrifugation. Relative β-estradiol serum levels were detected by the Mouse/Rat Estradiol ELISA-Kit (SKU: ES180S-100, Calbiotech, Spring Valley, CA USA) according to manufacturer’s instructions.

### Cell culture and IFN-γ treatment of embryonic fibroblasts

MEFs from C57BL/6N females that had been mated with C57BL/6N PA28αOE heterozygote males, were isolated at E13.5 as described [61], with the following exceptions: embryos were isolated individually, heads were used for genotyping and the trypsin treatment was for 45 min in 0.05% trypsin–EDTA solution with 1% chicken serum (Gibco, Thermo Fisher Scientific, Gothenburg, Sweden) under gentle agitation. Cells were cultivated in DMEM (Dulbecco’s modified Eagle’s medium, Thermo Fisher Scientific) supplemented with 10% fetal bovine serum (FBS), 1% Penicillin/streptomycin and 1% non-essential amino acids at 37 °C under 5% CO2 and ambient oxygen. For positive control in the analysis of PA28αβ-dependent proteasome capacity, 150 U/mL recombinant mouse IFN-γ (Thermo Fisher Scientific) was added to the culture media 24 h prior harvest.

### Proteasome capacity assays

PA28–20S or 20S proteasome capacity was analyzed as previously described [32] with some modifications. Cells were lysed in 25 mM Tris/HCl (pH 8.3) by 4 cycles of high-speed centrifugation (20,000 g) and resuspension at 4 °C, cell debris was removed by centrifugation at 5000 g for 10 min and protein concentration was determined using the BCA Protein Assay kit (Pierce, Thermo Fisher Scientific). The chemotryptic activity was assayed by hydrolysis of the fluorogenic peptide succinyl-Leu-Leu-Val-Tyr-7-amino-4-methylcoumarin (suc-LLVY-AMC; Calbiochem Merck-Millipore, Darmstadt, Germany). 10 µg total protein was incubated with 200 µM suc-LLVY-AMC in 50 mM Tris/HCl (pH 8.3) and 0.5 mM DTT for PA28–20S activity or 50 mM Tris/HCl (pH 8.3), 0.5 mM DTT and 0.02% SDS for 20S activity in a total volume of 100 µL; fluorescence was monitored using 390 nm excitation and 460 nm emission filters with free AMC as standard (Molekula Ltd., Gillingham, UK) and activity was determined as the slope of fluorescence over time divided by total protein. Protein levels in the assay were determined by SDS–PAGE, InstantBlueTM (Expedeon Ltd., Cambridge UK) staining, and analysis using the Odyssey infrared imaging system and software (LI-COR Biosciences). Activity upon proteasome inhibition with 5 µM epoxomicin (Sigma-Aldrich, Stockholm, Sweden) is considered non-specific/background activity. Epoxomicin inhibited the PA28–20S proteasome capacity to 70 ± 9% (mean ± SD) of WT, 77 ± 10% of PA28αOE, 98.3% of untreated MEFs and 99.8% of interferon-γ treated MEFs; and epoxomicin inhibited the 20S proteasome capacity to 94 ± 3% of WT, 95 ± 3% of PA28αOE, 85% of untreated MEFs and 81% of interferon-γ treated MEFs.

### Luciferase aggregation prevention

Luciferase aggregation prevention capacity was analyzed as previously described [62] with some modifications. To increase the number of n in the analysis, hippocampi from females of similar age (5–6 months) of the C57BL/6N background, 6 WT and 6 PA28αOE, were included to the 4 WT and 3 PA28αOE hippocampi from the C57BL/6N × BALB/c F2 hybrids. Right hippocampi were lysed in extraction buffer (25 mM Tris/HCl, 100 mM NaCl, 5 mM MgCl_2_, 1 mM ATP, and 5% glycerol, pH 7.4) by 4 cycles of high-speed centrifugation (20,000 g) and resuspension at 4 °C. Cell debris was removed by centrifugation at 5000 g for 10 min and 1 mM DTT was added after an aliquot was set aside for protein concentration determination with the BCA Protein Assay kit (Pierce, Thermo Fisher). Heat-sensitive luciferase (200 nM; L9506; Sigma-Aldrich) was heat-denatured at 42 °C in 50 mM Tris pH 7.6, 2 mM EDTA, in the presence of 4.5 µg protein extracts or corresponding volume of extraction buffer. Aggregation of luciferase was determined as light scattering at 340 nm at 42 °C. At around 80% of maximum, the increase in turbidity of the positive control (without protein extract) started to plateau, and the closest time point was chosen for analysis (40 min in the experiments on hybrid hippocampal extracts and 20 min at 42 °C in the experiments with C57). The turbidity of the positive control was considered maximum aggregation (100%). Turbidity of the negative control with no addition of heat-sensitive luciferase did not change over time and was considered background. Luciferase aggregation prevention capacity was calculated as percentage of non-aggregated luciferase. Extracts that had been incubated at 99 °C for 45 min served as negative control to the cell extract and did not prevent aggregation.

### Statistical analysis

Comparisons between two groups were performed with unpaired *t* test assuming two-tailed distribution and equal variances and differences were considered significant at *P* < 0.05. Statistical analysis of the activity box corner time day 2 (Fig. 3a) by two-way ANOVA repeated measurements followed by Sidak multiple comparisons test and of the shuttle box PAT (Fig. 3b) was done by Mantel–Cox survival test; both in GraphPad Prism and the null hypothesis was rejected at the 0.05 level.

## Additional files

**Additional file 1.** The raw data used to produce Fig. 1.

**Additional file 2.** Physiological parameters of WT and PA28αOE F2 C57BL/6NxBALB/c mice.

**Additional file 3.** The cellular immune profiles of PA28αOE and WT mice.

**Additional file 4.** Blood glucose and insulin response in oral glucose tolerance test (OGTT) of PA28αOE and WT mice.

**Additional file 5.** The raw data used to produce Fig. 2.

**Additional file 6.** The raw data used to produce Fig. 3.

**Additional file 7.** Tail-flick pain tolerance analysis of PA28αOE.

**Additional file 8.** Cognitive behavior of male PA28αOE.

**Additional file 9.** Hippocampal neuronal markers and serum estrogen levels of PA28αOE and WT mice.

**Additional file 10.** The raw data used to produce Fig. 4a and 4c.

**Additional file 11.** PA28-dependent proteasome activity of PA28αOE and WT MEFs.

**Additional file 12.** The raw data used to produce Additional file 11.

**Additional file 13.** The raw data used to produce Fig. 4b, 4d, 4e and Additional file 9.

**Additional file 14.** Sequences of primers used for real-time quantitative (qPCR) analysis.

## Abbreviations

GFPdgn: GFP fused to degron CL1, reporter gene
H_2_O_2_: hydrogen peroxide
LTP: long-term potentiation
MHC-I: major histocompatibility complex I
MEFs: mouse embryonic fibroblasts
PA28αOE: PA28α overexpression mouse model
WT: wildtype
