# Supplementary material for: PA28αβ overexpression enhances learning and memory of female mice without inducing 20S proteasome activity
Source: BMC Neurosci. 2018 Nov 6;19:70. doi: 10.1186/s12868-018-0468-2 (PMC6218978; doi:10.1186/s12868-018-0468-2)
Supplement: Supplementary file 1 — Additional file 1. The raw data used to produce Fig. 1. [file 12868_2018_468_MOESM1_ESM.pdf]

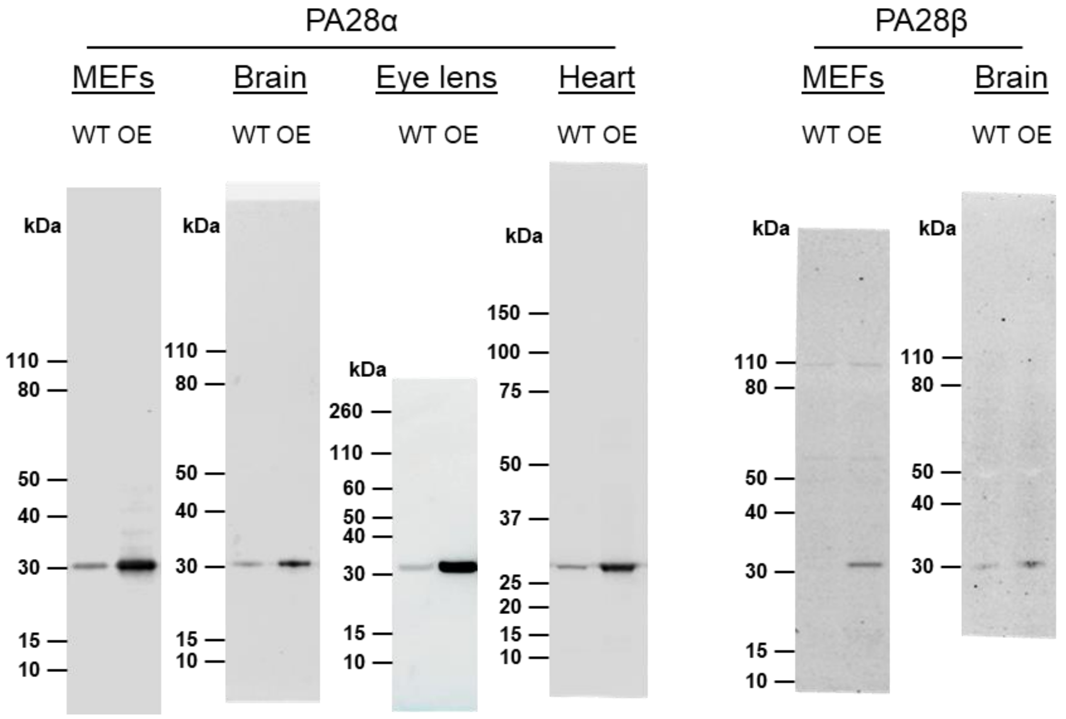

animal	Ref gene: 36b4		PA28alpha				PA28betav1				PA28betav2			
	CT	2^ (35)	CT	2^ (35)	N* 36b4	N* WT mean	CT	2^ (35)	N* 36b4	N* WT mean	CT	2^ (35)	N* 36b4	N* WT mean
**WT C57:1**														
Repl 1	28,8	74,4	32,2	6,94	0,09	1,22	31,6	10,4	0,140	1,296	29,4	49,5	0,664	1,47
Repl 2	28,7	79,2	32,1	7,60	0,10	1,25	32,2	7,16	0,090	0,836	29,6	41,1	0,519	1,15
Repl 3	29,0	62,6	32,4	6,15	0,10	1,28	31,6	10,3	0,164	1,518	29,4	49,2	0,785	1,73
**OE C57:1**														
Repl 1	27,0	255	27,7	161	0,63	8,26	30,9	16,8	0,066	0,608	28,9	70,6	0,277	0,61
Repl 2	27,2	222	28,3	107	0,48	6,30	31,0	16,4	0,074	0,684	28,6	87,4	0,394	0,87
Repl 3	27,1	240	27,7	154	0,64	8,37	30,9	16,7	0,069	0,641	28,7	80,2	0,334	0,74
**WT C57:2**														
Repl 1	27,9	136	32,4	6,04	0,04	0,58	30,8	17,9	0,131	1,214	29,2	57,4	0,421	0,93
Repl 2	28,0	132	34,1	1,91	0,01	0,19	31,1	14,6	0,111	1,024	29,1	59,3	0,449	0,99
Repl 3	28,1	116	32,7	4,96	0,04	0,56	31,0	16,1	0,140	1,290	29,0	62,3	0,540	1,19
**OE C57:2**														
Repl 1	27,3	210	27,3	212	1,01	13,19	30,8	18,5	0,088	0,818	29,0	64,9	0,309	0,68
Repl 2	27,4	191	27,9	133	0,69	9,07	30,7	19,5	0,102	0,943	28,8	75,2	0,394	0,87
Repl 3	27,5	182	27,4	195	1,07	13,94	30,6	20,7	0,113	1,049	28,7	79,2	0,434	0,96
**OE C57:3**														
Repl 1	32,3	6,33	*nd*	-	-	-	34,7	1,27	0,201	1,860	33,8	2,35	0,371	0,82
Repl 2	32,6	5,27	33,6	2,65	0,50	6,55	34,8	1,18	0,223	2,061	33,8	2,24	0,424	0,94
Repl 3	32,9	4,38	34,6	1,33	0,30	3,95	34,6	1,32	0,302	2,788	32,9	4,21	0,961	2,12
**WT C57:3**														
Repl 1	28,0	127	31,2	13,8	0,11	1,42	32,1	7,66	0,060	0,557	31,0	16,3	0,129	0,28
Repl 2	27,9	139	31,3	13,3	0,10	1,24	32,2	6,82	0,049	0,453	30,4	24,6	0,177	0,39
Repl 3	28,8	74,1	32,2	7,18	0,10	1,26	32,3	6,51	0,088	0,811	30,1	29,3	0,395	0,87

Additional file 1: Raw data to figure 1. Upper: Full-length blots of cropped blots shown in Fig. 1b. Protein extracts from eye lens were separated on a shorter gel. Lower: Raw data table for mRNA levels of PA28α and PA28β shown in Fig. 1c). N*: Normalized to, median values of replicate samples are used as a representative for the biological replicate.
