# Supplementary material for: PA28αβ overexpression enhances learning and memory of female mice without inducing 20S proteasome activity
Source: BMC Neurosci. 2018 Nov 6;19:70. doi: 10.1186/s12868-018-0468-2 (PMC6218978; doi:10.1186/s12868-018-0468-2)
Supplement: Supplementary file 2 — Additional file 2. Physiological parameters of WT and PA28αOE F2 C57BL/6NxBALB/c mice. [file 12868_2018_468_MOESM2_ESM.pdf]

Additional file 2

Physiological parameters of WT and PA28αOE F2 C57BL/6NxBALB/c mice

	Body temp (°C)	Body weight (g)	Body length (cm)	Fat mass (g)	Lean mass (g)	Bone Density (mg/cm^2)
WT	38.4 ± 0.2	34.6 ± 1.6	10.3 ± 0.1	11.0 ± 0.8	20.0 ± 0.6	56.6 ± 0.8
OE	38.3 ± 0.2	30.2 ± 1.6	10.2 ± 0.1	8.6 ± 1.2	18.4 ± 0.5	54.8 ± 1.2
p-value	*0.74*	*0.09*	*0.26*	*0.11*	*0.09*	*0.24*

Rectal core body temperature and body composition measured by Dual energy X-ray absorptiometry (DEXA) of WT and PA28αOE 8 month-old female F2 C57BL/6NxBALB/c mice. Data represent mean±SEM, p-values were obtained from unpaired two-tailed t-test assuming two-tailed distribution and equal variances (n=9 for WT; n=6 for OE).

Raw data:

**Mouse ID** WT	Body temp (°C)	Body weight (g)	Body length (cm)	Fat mass (g)	Lean mass (g)	Bone Density (mg/cm^2)
226	37,7	41,0	10,9	14,9	21,8	57,0
228	37,9	33,9	10,3	10,1	20,3	61,1
341	37,9	32,6	10,1	10,8	18,3	53,3
384	38,8	29,7	10,2	8,9	18,1	54,1
342	38,5	38,6	10,4	12,9	21,6	58,9
459	38,9	29,0	10,0	8,1	18,1	59,1
230	38,1	33,7	10,4	9,8	20,0	56,6
406	38,9	41,7	10,5	14,7	22,9	55,6
419	38,7	30,9	10,3	9,2	19,0	55,7
PA28αOE						
268	38,9	25,2	10,1	4,0	18,1	56,1
267	37,7	35,6	10,5	11,4	20,7	54,1
382	37,8	32,1	10,3	10,8	17,6	57,4
381	37,8	31,7	10,2	9,9	18,7	56,4
270	38,5	26,4	10,1	6,1	17,4	57,8
272	39,0	30,4	10,0	9,6	18,4	50,1
