# Supplementary material for: PA28αβ overexpression enhances learning and memory of female mice without inducing 20S proteasome activity
Source: BMC Neurosci. 2018 Nov 6;19:70. doi: 10.1186/s12868-018-0468-2 (PMC6218978; doi:10.1186/s12868-018-0468-2)
Supplement: Supplementary file 3 — Additional file 3. The cellular immune profiles of PA28αOE and WT mice. [file 12868_2018_468_MOESM3_ESM.pdf]

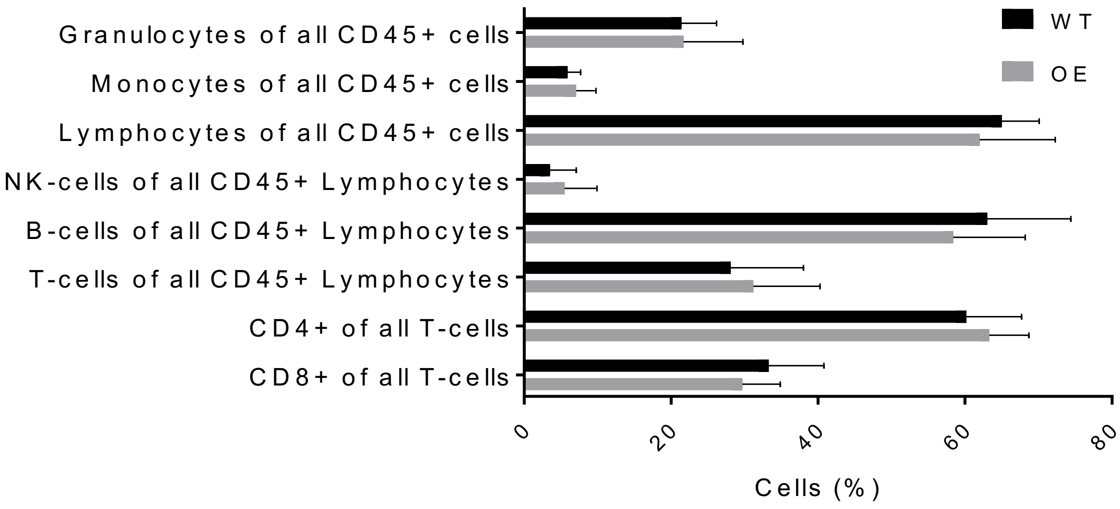


**Additional file 3: The cellular immune profiles of PA28αOE and WT female mice do not differ.** Immuno-phenotyping of peripheral blood by flow cytometric cell sorting: from all leucocytes (CD45+), granularity distinguished monocytes, granulocytes and lymphocytes; monocytes were validated with CD4/80+; the lymphocyte population was divided into NK cells (CD161c+), B cells (CD19+) and T cells (CD3+, CD161-); T cells were separated into CD4+ and CD8+ fractions. Values are mean±SD; nPA28αOE=6 and nWT=9. Raw data to the figure is found below.

| | % Granulocytes of all CD45+ cells | % Lymphocytes of all CD45+ cells | % B-cells of all CD45+ Lymphocytes | % NK-cells of all CD45+ Lymphocytes | % T-cells of all CD45+ Lymphocytes | % CD4+ of all T-cells | % CD8+ of all T-cells | % Monocytes of all CD45+ cells |
|---|---|---|---|---|---|---|---|---|
| Standard | 75 | 8,33 | 50 | 0 | 0 | 0 | 0 | 0 |
| WT | | | | | | | | |
| 226 | 24,5 | 61 | 76 | 1,59 | 15,2 | 74,9 | 18,5 | 7,13 |
| 228 | 16,3 | 65,7 | 84,3 | 1,81 | 12,4 | 51,2 | 43,9 | 9,72 |
| 341 | 23,3 | 64,7 | 65,8 | 10,4 | 20,5 | 63,3 | 31,6 | 5,7 |
| 342 | 17,5 | 70,6 | 62,9 | 1,03 | 29,7 | 64,1 | 30,1 | 6,18 |
| 406 | 17,5 | 69,7 | 58,5 | 5,22 | 32,5 | 57,9 | 37,6 | 4,51 |
| 419 | 28 | 57,2 | 53,2 | 8,33 | 34 | 64,9 | 31,1 | 5,8 |
| 459 | 16,3 | 72,1 | 62,6 | 0,5 | 31,8 | 52,1 | 39,2 | 4,4 |
| 230 | 28,0 | 60,2 | 47,0 | 2,2 | 41,1 | 53,5 | 38,5 | 4,0 |
| 384 | 21,3 | 64 | 57 | 0,81 | 35,7 | 60,3 | 29,3 | 5,8 |
| PA28OE | | | | | | | | |
| 267 | 19,5 | 64,9 | 66,7 | 7,9 | 21,8 | 61,2 | 33,1 | 6,3 |
| 268 | 25,1 | 58,8 | 59,6 | 2,8 | 31,6 | 55,1 | 38,5 | 7,4 |
| 270 | 19,6 | 55,2 | 64,8 | 2,6 | 27,3 | 69,4 | 25,3 | 11,6 |
| 272 | 20,5 | 62,0 | 41,8 | 0,9 | 47,9 | 60,5 | 26,2 | 7,0 |
| 381 | 10,3 | 80,6 | 65,5 | 6,1 | 26,7 | 65,0 | 28,6 | 3,5 |
| 382 | 35,1 | 50,6 | 52,0 | 12,7 | 31,6 | 68,4 | 26,7 | 6,9 |
