# Supplementary material for: PA28αβ overexpression enhances learning and memory of female mice without inducing 20S proteasome activity
Source: BMC Neurosci. 2018 Nov 6;19:70. doi: 10.1186/s12868-018-0468-2 (PMC6218978; doi:10.1186/s12868-018-0468-2)
Supplement: Supplementary file 4 — Additional file 4. Blood glucose and insulin response in oral glucose tolerance test (OGTT) of PA28αOE and WT mice. [file 12868_2018_468_MOESM4_ESM.pdf]

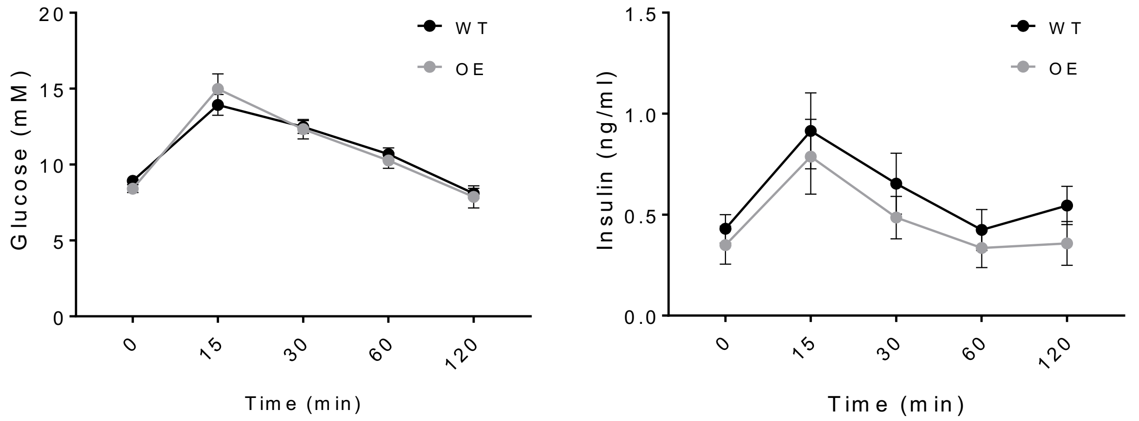

Mouse ID	Glucose					Insulin				
WT	BL	15min	30min	60min	120min	BL	15min	30min	60min	120min
226	8,3	16,4	11,6	11,1	9,8	0,892	2,123	1,626	1,095	1,149
228	9,8	13,2	10,7	12,3	7	0,262	1,377	0,613	0,498	0,452
230	7,3	10,7	10,8	9,9	6,5	0,288	0,427	0,436	0,322	0,510
231	7,4	15	13,7	9,1	7,8	0,343	0,623	0,571	0,382	0,725
341	9,5	17	11,9	11,5	8	0,568	1,271	1,417	0,852	0,905
342	9,2	16	12,7	12,8	8,7	0,199	1,147	0,362	0,256	0,212
406	9,9	14,5	14,9	11,2	7,8	0,622	0,521	0,397	0,185	0,282
384	10,4	12	12	9,3	8,9	0,565	1,099	0,591	0,415	0,477
459	8,5	11,6	13,4	10,2	9,3	0,238	0,185	0,199	0,093	0,265
419	8,9	12,9	13,1	9,5	7,2	0,322	0,375	0,322	0,146	0,477
PA28αOE										
267	8,2	18,4	13,4	8,5	7	0,458	1,165	0,599	0,488	0,469
268	7,7	12,5	14,8	10,5	8,3	0,107	0,382	0,352	0,179	0,182
270	7,9	12,1	12,2	10,9	7,2	0,523	0,773	0,650	0,424	0,531
272	8,8	15,3	10,7	9,8	5,7	0,489	1,328	0,839	0,668	0,729
381	8,5	16,8	10,8	9,7	8	0,512	0,927	0,349	0,243	0,229
382	9,4	14,8	12,1	12,2	11	0,004	0,146	0,124	0,006	0,004

Additional file 4: PA28αOE and WT female mice display similar blood glucose and insulin response in oral glucose tolerance test (OGTT). Blood glucose and insulin were measured before glucose dosing (0) and after 15, 30, 60 and 120 minutes to follow return to baseline. Values are mean±SEM; $n_{PA28\alpha OE}=6$ and $n_{WT}=10$. Data is plotted above and presented in full below.
