# Supplementary material for: PA28αβ overexpression enhances learning and memory of female mice without inducing 20S proteasome activity
Source: BMC Neurosci. 2018 Nov 6;19:70. doi: 10.1186/s12868-018-0468-2 (PMC6218978; doi:10.1186/s12868-018-0468-2)
Supplement: Supplementary file 5 — Additional file 5. The raw data used to produce Fig. 2. [file 12868_2018_468_MOESM5_ESM.pdf]

Forced Swim test

Mouse ID			
WT	Passive %	Active %	Distance traveled (cm)
226	93	7	920
228	87	13	199
341	96	4	1023
384	60	40	1689
342	91	9	756
459	64	36	1385
230	86	14	986
406	87	13	1269
419	85	15	660
PA28αOE			
268	95	5	371
267	32	68	2580
382	74	26	1826
381	79	21	788
270	56	44	1765
272	5	95	3070

ZeroMaze

Mouse ID		
WT	Activity	Open (s)
226	141	0
228	117	0,1
341	309	9,3
384	123	0
342	241	0,5
459	159	3,2
230	74	0
406	221	33,9
419	103	0,5
PA28αOE		
268	302	8,5
267	135	21,7
382	190	0,6
381	268	0,6
270	281	1,3
272	122	7

Additional file 5: Raw data to figure 2.
