# Supplementary material for: PA28αβ overexpression enhances learning and memory of female mice without inducing 20S proteasome activity
Source: BMC Neurosci. 2018 Nov 6;19:70. doi: 10.1186/s12868-018-0468-2 (PMC6218978; doi:10.1186/s12868-018-0468-2)
Supplement: Supplementary file 6 — Additional file 6. The raw data used to produce Fig. 3. [file 12868_2018_468_MOESM6_ESM.pdf]

Activity box: Locomotion

Mouse ID	Day 1						Day 2					
WT	10min	20min	30min	40min	50min	60min	10min	20min	30min	40min	50min	60min
226	640	330	234	319	206	143	406	338	136	225	110	62
228	419	221	121	86	79	174	361	129	227	120	157	18
230	784	609	694	618	465	480	857	476	552	408	160	304
231	727	388	464	529	449	400	637	399	330	330	399	360
341	598	452	353	490	574	373	711	741	503	611	455	399
342	913	619	545	455	364	464	701	615	381	795	405	652
406	314	354	426	469	536	21	548	547	315	639	436	334
384	708	560	369	425	387	76	822	378	408	406	333	346
459	778	350	377	233	291	244	1415	847	985	895	944	756
419	266	7	64	174	219	191	465	107	290	128	35	89
PA28αOE												
267	355	267	335	238	192	201	831	293	243	365	243	56
268	566	225	261	206	233	251	627	496	523	381	280	46
270	573	426	318	426	263	240	479	279	235	2	1	0
272	483	539	589	584	337	577	403	215	283	281	171	214
381	815	791	579	546	621	485	340	116	193	3	47	146
382	416	295	311	378	302	429	640	328	485	504	564	498

Activity box: Corner time

Mouse ID	Day 1						Day 2					
WT	10min	20min	30min	40min	50min	60min	10min	20min	30min	40min	50min	60min
226	12	4	5	43	79	300	20	87	260	155	364	472
228	61	147	264	264	229	109	174	258	264	391	334	571
230	24	35	25	48	66	136	104	60	41	41	65	142
231	63	65	108	82	178	154	152	222	250	346	155	131
341	54	29	5	20	8	12	58	77	51	15	14	25
342	30	43	51	25	55	54	18	8	6	13	4	21
406	112	130	165	200	139	532	106	129	336	78	161	254
384	75	41	35	45	163	461	42	35	118	51	22	16
459	24	26	20	47	64	71	29	46	45	58	27	29
419	369	591	550	474	387	243	65	34	40	227	214	265
PA28αOE												
267	120	154	93	235	280	384	33	89	163	126	260	533
268	24	52	31	20	94	200	125	217	265	350	386	566
270	15	53	58	53	104	63	215	192	263	596	600	600
272	97	55	34	44	81	96	76	257	210	236	238	194
381	63	51	105	72	72	105	372	449	440	591	572	504
382	118	58	60	66	92	24	109	129	47	17	44	24

Activity box: Rearings

Mouse ID	Day 1						Day 2					
WT	**10min**	**20min**	**30min**	**40min**	**50min**	**60min**	**10min**	**20min**	**30min**	**40min**	**50min**	**60min**
226	322	145	167	199	151	188	135	133	122	178	150	105
228	120	122	148	125	134	151	128	129	159	126	176	49
230	187	287	351	319	338	338	152	210	205	177	206	118
231	207	113	164	298	281	264	155	135	176	190	217	217
341	65	114	155	154	168	218	198	246	265	394	346	371
342	320	325	380	331	361	347	212	354	320	366	303	372
406	143	160	194	231	284	23	185	288	219	358	296	213
384	95	11	28	58	62	114	112	91	139	126	79	132
459	187	177	126	104	152	230	373	470	488	555	622	609
419	123	90	80	94	94	26	129	65	152	124	42	82
PA28αOE												
267	305	287	349	256	255	224	185	144	122	149	133	18
268	68	80	90	100	71	70	158	178	216	137	141	29
270	121	120	120	175	149	81	242	169	201	7	5	0
272	105	184	190	184	132	133	66	64	69	130	75	95
381	316	481	470	531	566	408	35	32	50	7	12	33
382	96	54	89	96	84	159	75	72	166	250	280	262

Passive avoidance test

Day 1

**Mouse ID** WT	**Respcs**	**Elapstime**	**Adapt**
226	25,6	88	60,0
228	49,7	112,1	60,0
341	7,8	70,2	60,0
384	12,9	75,3	60,0
342	42,2	104,6	60,0
459	29,9	92,3	60,0
230	111,3	173,7	60,0
406	95,5	157,9	60,0
419	159,1	221,5	60,0
PA28αOE			
268	9,9	72,3	60,0
267	26,1	88,5	60,0
382	22,7	85,1	60,0
381	40,4	102,8	60,0
270	26,5	88,9	60,0
272	67,9	130,3	60,0

Day 2

**Mouse ID** WT	**Respcs**	**Elapstime**	**Adapt**
226	84,5	146,9	60,0
228	34,7	97,1	60,0
341	19,3	81,7	60,0
384	300	360	60,0
342	300	360	60,0
459	32,7	95,1	60,0
230	249	311,4	60,0
406	21,9	84,3	60,0
419	91,3	153,7	60,0
PA28αOE			
268	300	360	60,0
267	300	360	60,0
382	300	360	60,0
381	300	360	60,0
270	300	360	60,0
272	300	360	60,0

Additional file 6: Raw data to figure 3
