# Supplementary material for: PA28αβ overexpression enhances learning and memory of female mice without inducing 20S proteasome activity
Source: BMC Neurosci. 2018 Nov 6;19:70. doi: 10.1186/s12868-018-0468-2 (PMC6218978; doi:10.1186/s12868-018-0468-2)
Supplement: Supplementary file 7 — Additional file 7. Tail-flick pain tolerance analysis of PA28αOE. [file 12868_2018_468_MOESM7_ESM.pdf]

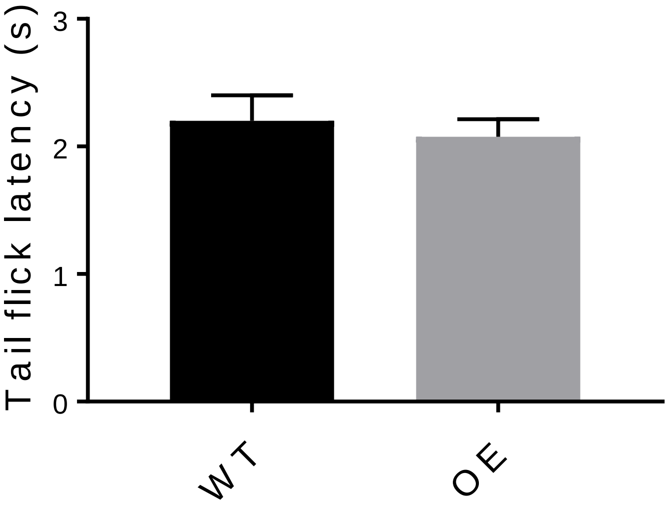

Additional figure 7. PA28αOE female mice are not more pain sensitive. In the tail flick test, reaction time to a pain stimulus (Water 52°C) is considered a measurement of pain sensitivity. No difference in pain sensitivity could be detected between C57BL/6N WT and PA28αOE female mice. Values are mean±SEM, $n_{PA28αOE}$=13 n_{WT}=10. Raw data are presented below.

Animal nr:	Genotype	Age	Tail flick latency (s)
581	PA28αOE	2 ms, 3wks	2
582	PA28αOE	2 ms, 3wks	3
583	WT	2 ms, 3wks	2
591	WT	2 ms, 1wks	2
592	PA28αOE	2 ms, 1wks	2
593	PA28αOE	2 ms, 1wks	2
597	WT	2 ms, 1wks	2
598	PA28αOE	2 ms, 1wks	2
599	WT	2 ms, 1wks	1
604	WT	2 ms	3
605	WT	2 ms	3
606	PA28αOE	2 ms	2
611	PA28αOE	1 m, 3 wks	2
612	PA28αOE	1 m, 3 wks	1
613	WT	1 m, 3 wks	3
614	PA28αOE	1 m, 3 wks	2
618	PA28αOE	1 m, 3 wks	3
619	WT	1 m, 3 wks	2
620	WT	1 m, 3 wks	2
621	WT	1 m, 3 wks	2
622	PA28αOE	1 m, 3 wks	2
623	PA28αOE	1 m, 2 wks	2
624	PA28αOE	1 m, 2 wks	2
