# Supplementary material for: PA28αβ overexpression enhances learning and memory of female mice without inducing 20S proteasome activity
Source: BMC Neurosci. 2018 Nov 6;19:70. doi: 10.1186/s12868-018-0468-2 (PMC6218978; doi:10.1186/s12868-018-0468-2)
Supplement: Supplementary file 8 — Additional file 8. Cognitive behavior of male PA28αOE. [file 12868_2018_468_MOESM8_ESM.pdf]

Shuttle box test on male PA28αOE and WT mice

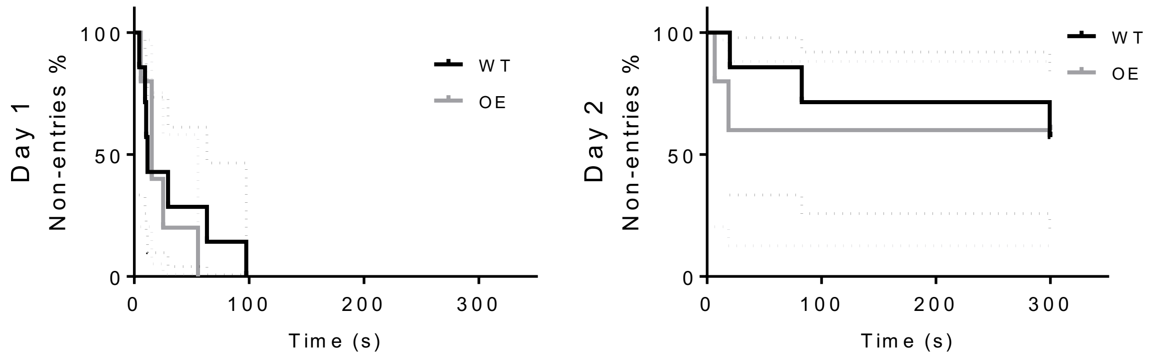

Forced swim test on male PA28αOE and WT mice

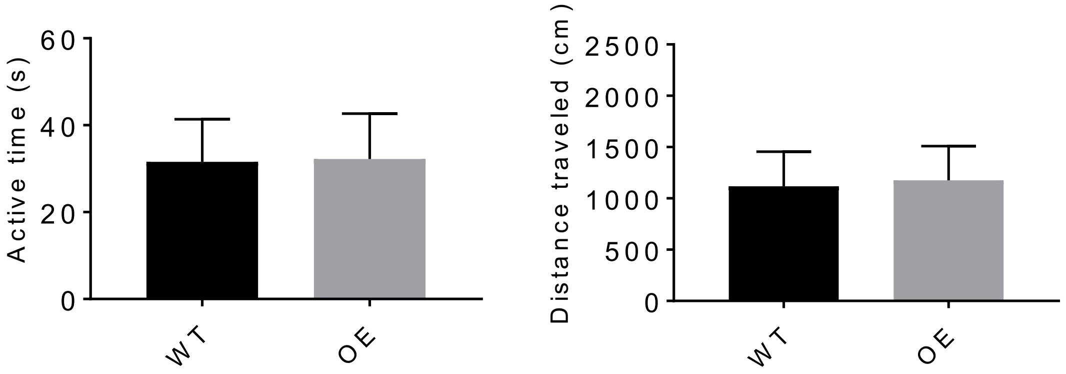

Additional file 8: There are no differences in cognitive behavior comparing male PA28αOE and WT mice, and thus the findings on female PA28αOE and WT mice are gender specific.

Upper plots: Shuttle box passive avoidance test on male mice, time of entry on day 1 (left) and day 2 (right). There is no difference between PA28αOE and WT in re-entering the compartment; n(WT)=7, n(OE)=5. Maximum assay time was 300 s (i.e. no entry=300s). Dashed lines correspond to 95% confidence interval.

Lower plots: Forced swim test, active time (left) and distance travelled (right). There is no difference between PA28αOE and WT in depressive-like behavior, which a reduction in these parameters would be a sign of. Values are mean±SEM; n(WT)=7, n(OE)=5.

Next page: Activity Box measurements of exploratory behavior; locomotion, rearing and corner time in novel (day 1) and acquainted (day 2) environment. There are no indications of enhanced habituation in the male PA28αOE mice compared to WT. Values are mean±SEM.; day1 n(WT)=7, n(OE)=4; day2 n(WT)=7, n(OE)=5.

Pages thereafter: Raw data for male behavioral tests.

Activity box analysis on male PA28αOE and WT mice

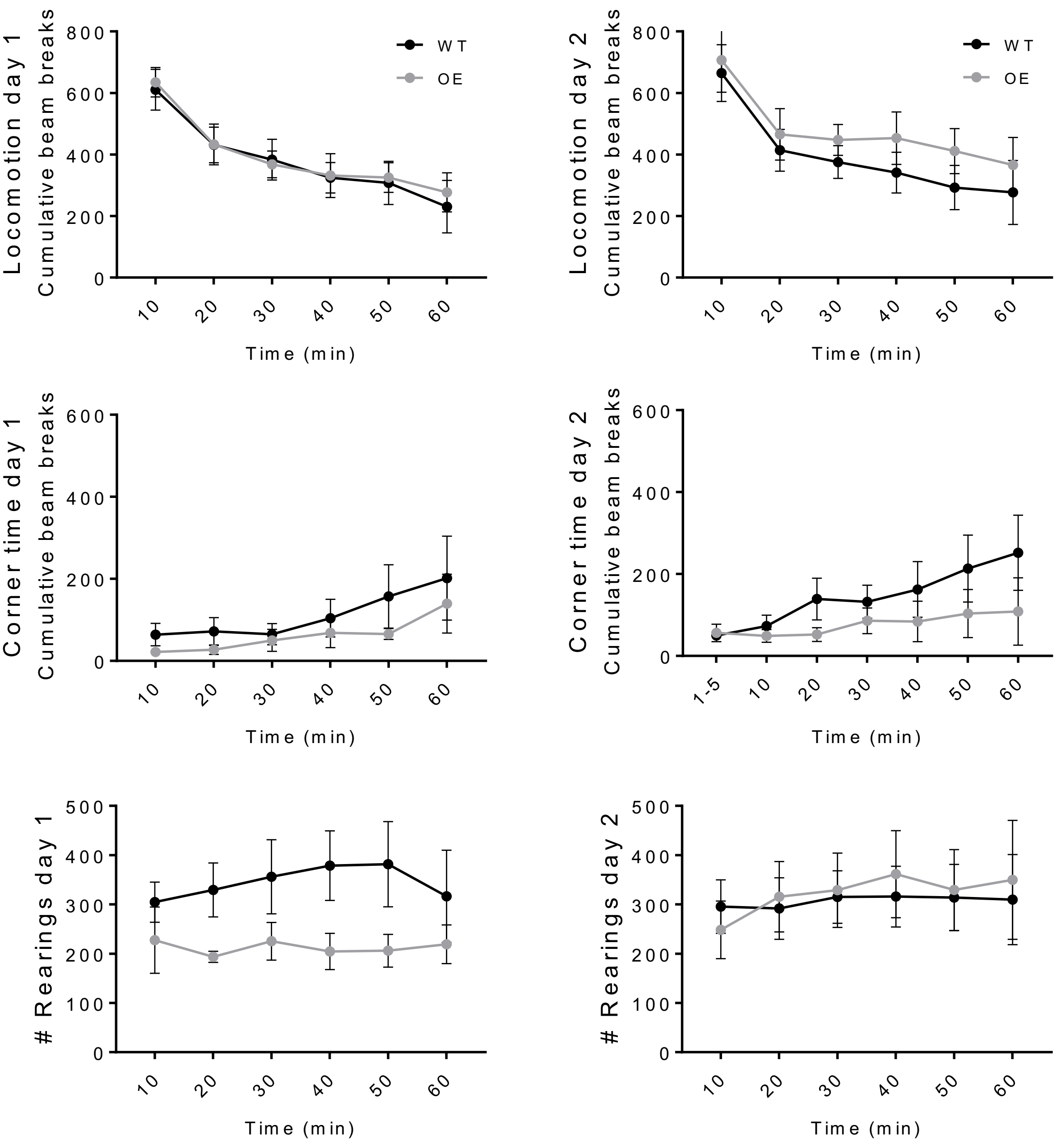

Raw data for male behavioral tests:

Passive avoidance test

Day 1

**Mouse ID** WT	**Respcs**	**Elapstime**	**Adapt**
387	11,6	74	60,0
388	97,6	160	60,0
392	29,5	91,9	60,0
184	4,3	66,7	60,0
185	63,3	125,7	60,0
187	10,4	72,8	60,0
599	9,5	71,9	60,0
PA28αOE			
147	15,2	77,6	60,0
148	25,1	87,5	60,0
151	5,8	68,2	60,0
598	55,5	117,9	60,0
601	15,2	77,6	60,0

Day 2

**Mouse ID** WT	**Respcs**	**Elapstime**	**Adapt**
387	300	360	60,0
388	299,3	361,7	60,0
392	300	360	60,0
184	82,7	145,1	60,0
185	20	82,4	60,0
187	300	360	60,0
599	300	360	60,0
PA28αOE			
147	300	360	60,0
148	300	360	60,0
151	18,7	81,1	60,0
598	300	360	60,0
601	6,8	69,2	60,0

Forced swim test

**Mouse ID** WT	Passive %	Active %	Distance traveled (cm)
387	27	73	2394
388	99	1	662
392	90	10	430
184	57	43	376
185	80	20	1227
187	46	54	2315
599	80	20	422
PA28αOE			
147	89	11	906
148	29	71	2262
151	70	30	1608
598	83	17	592
601	68	32	512

Activity box

Locomotion

Mouse ID	Day 1						Day 2					
WT	**10min**	**20min**	**30min**	**40min**	**50min**	**60min**	**10min**	**20min**	**30min**	**40min**	**50min**	**60min**
387	915	603	555	383	486	440	957	604	533	531	386	478
388	584	434	323	169	45	0	480	234	284	104	23	23
392	363	264	231	233	149	4	499	263	327	273	258	97
184	616	443	396	353	276	365	664	362	299	337	217	186
185	485	260	113	191	242	5	384	282	232	179	133	0
187	589	360	460	418	388	275	640	450	341	382	469	418
599	722	654	603	523	570	525	1028	701	612	584	563	737
PA28αOE												
147	578	562	496	382	414	463	440	413	405	280	331	323
148	532	397	328	439	236	223	677	371	388	635	366	308
151	734	509	341	384	403	245	820	458	405	315	251	161
598	695	263	306	122	248	178	557	302	391	353	427	334
601	-	-	-	-	-	-	1040	783	647	683	681	703

Corner time

Mouse ID	Day 1						Day 2					
WT	**10min**	**20min**	**30min**	**40min**	**50min**	**60min**	**10min**	**20min**	**30min**	**40min**	**50min**	**60min**
387	15	19	12	12	24	45	15	34	18	23	28	20
388	50	74	122	307	515	600	192	411	324	517	580	588
392	223	262	183	244	377	592	154	217	135	99	222	228
184	53	18	58	66	110	86	30	64	87	69	75	217
185	15	24	0	4	8	0	40	117	223	294	430	585
187	60	92	73	74	48	66	53	73	62	85	17	87
599	32	13	8	22	18	24	27	56	77	49	141	38
PA28αOE												
147	42	44	33	54	63	38	169	226	257	241	224	312
148	6	10	21	31	29	156	212	260	247	346	292	195
151	33	50	130	174	88	336	195	223	186	162	148	133
598	7	5	16	15	82	28	185	270	340	378	355	294
601	-	-	-	-	-	-	481	599	614	680	627	815

Rearing

Mouse ID	Day 1						Day 2					
WT	**10min**	**20min**	**30min**	**40min**	**50min**	**60min**	**10min**	**20min**	**30min**	**40min**	**50min**	**60min**
387	446	477	536	477	490	453	467	491	514	515	474	538
388	193	196	156	179	133	0	131	84	150	98	36	9
392	257	242	300	325	191	147	218	217	289	300	281	291
184	278	378	333	383	364	446	270	232	262	290	317	262
185	172	120	73	116	167	36	164	131	158	121	141	11
187	375	495	609	551	678	555	316	442	377	420	428	425
599	411	397	486	620	648	578	502	445	455	467	520	632
PA28αOE												
147	156	194	327	298	302	334	169	226	257	241	224	312
148	115	181	186	202	172	194	212	260	247	346	292	195
151	221	174	151	199	197	156	195	223	186	162	148	133
598	418	225	237	119	153	192	185	270	340	378	355	294
601	-	-	-	-	-	-	481	599	614	680	627	815
