# Supplementary material for: PA28αβ overexpression enhances learning and memory of female mice without inducing 20S proteasome activity
Source: BMC Neurosci. 2018 Nov 6;19:70. doi: 10.1186/s12868-018-0468-2 (PMC6218978; doi:10.1186/s12868-018-0468-2)
Supplement: Supplementary file 9 — Additional file 9. Hippocampal neuronal markers and serum estrogen levels of PA28αOE and WT mice. [file 12868_2018_468_MOESM9_ESM.pdf]

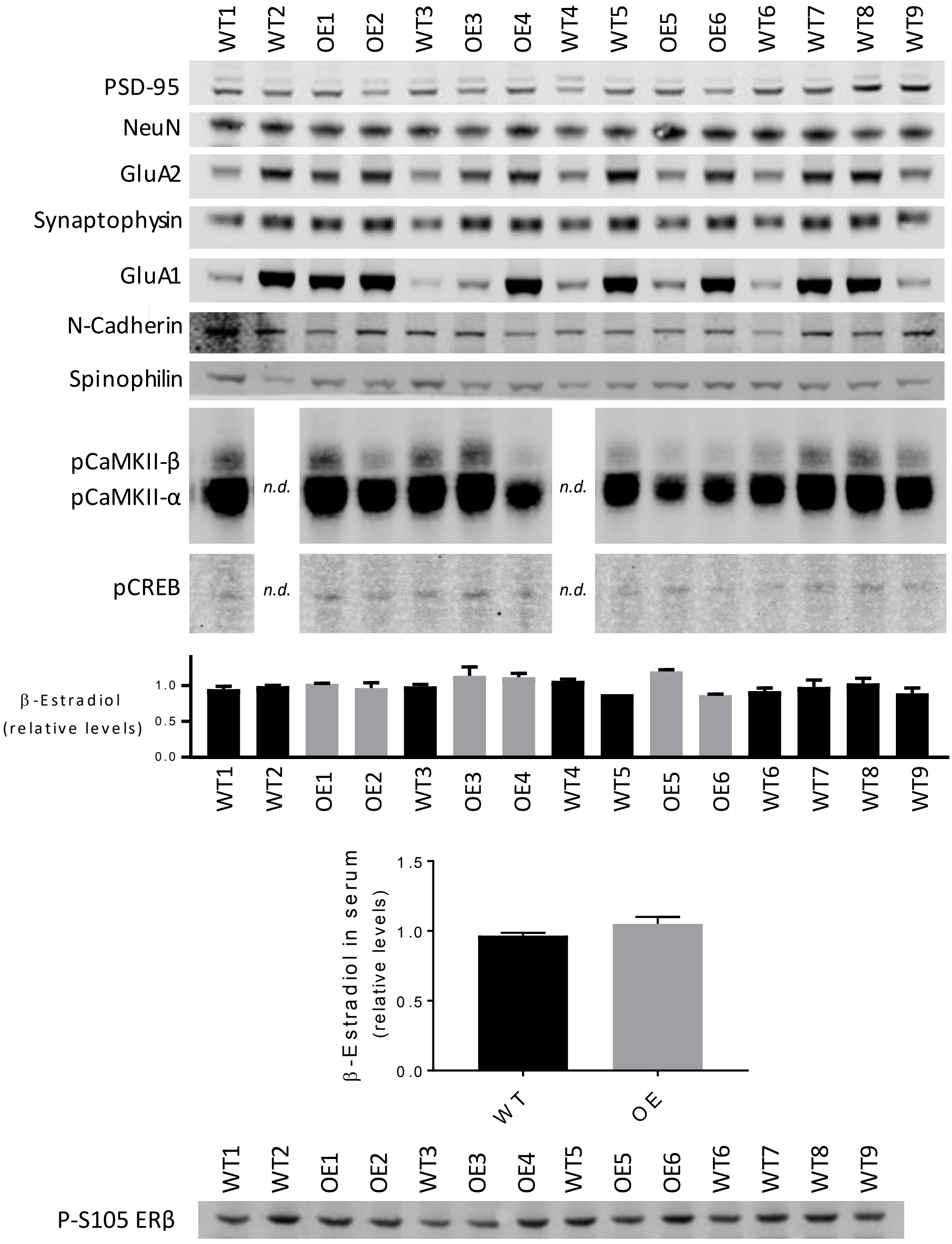

Additional file 9: There are no differences between PA28αOE and WT female mice in hippocampal neuronal markers and serum estrogen levels. Western analysis of the neuronal markers PSD-95, NeuN, GluA2, Synaptophysin, GluA1, N- Cadherin, Spinophilin T286-phosphorylated CaMKII, S133-phosphorylated CREB (upper blots) and S105-phosphorylated Estrogen receptor β (lower blot) in protein extracts from PA28αOE and WT left hippocampus. Quantification of β-Estradiol in serum from PA28αOE and WT, values are mean±SEM; $n_{PA28\alpha OE}$=6 and n_{WT}=9. The levels of GluA2, Synaptophysin, GluA1, and P-S105 ER β correlate to each other (correlation coefficients: $CC_{GluA2\text{-}Synphys}$: 0.93; $CC_{GluA2\text{-}GluA1}$=0.92; $CC_{GluA2\text{-}S105\text{-}ER\beta}$=0.72; $CC_{Synphys\text{-}GluA1}$=0.83; $CC_{Synphys\text{-}S105\text{-}ER\beta}$=0.61; $CC_{GluA1\text{-}S105\text{-}ER\beta}$=0.84). Raw data (full-length blots and β-Estradiol ELISA assay measurements and intra-assay CV:s) are included below, except for P-S105 ER β that is shown in Additional file 13.

Full-length blots of cropped blots shown on previous page. Some blots were cut in half prior immuno-detection. Identification of band corresponding to protein of interest was based on molecular weight and remaining bands were considered background detection. Due to limited amount of protein extract, the GluA1membrane was reused to detect Spinophilin.

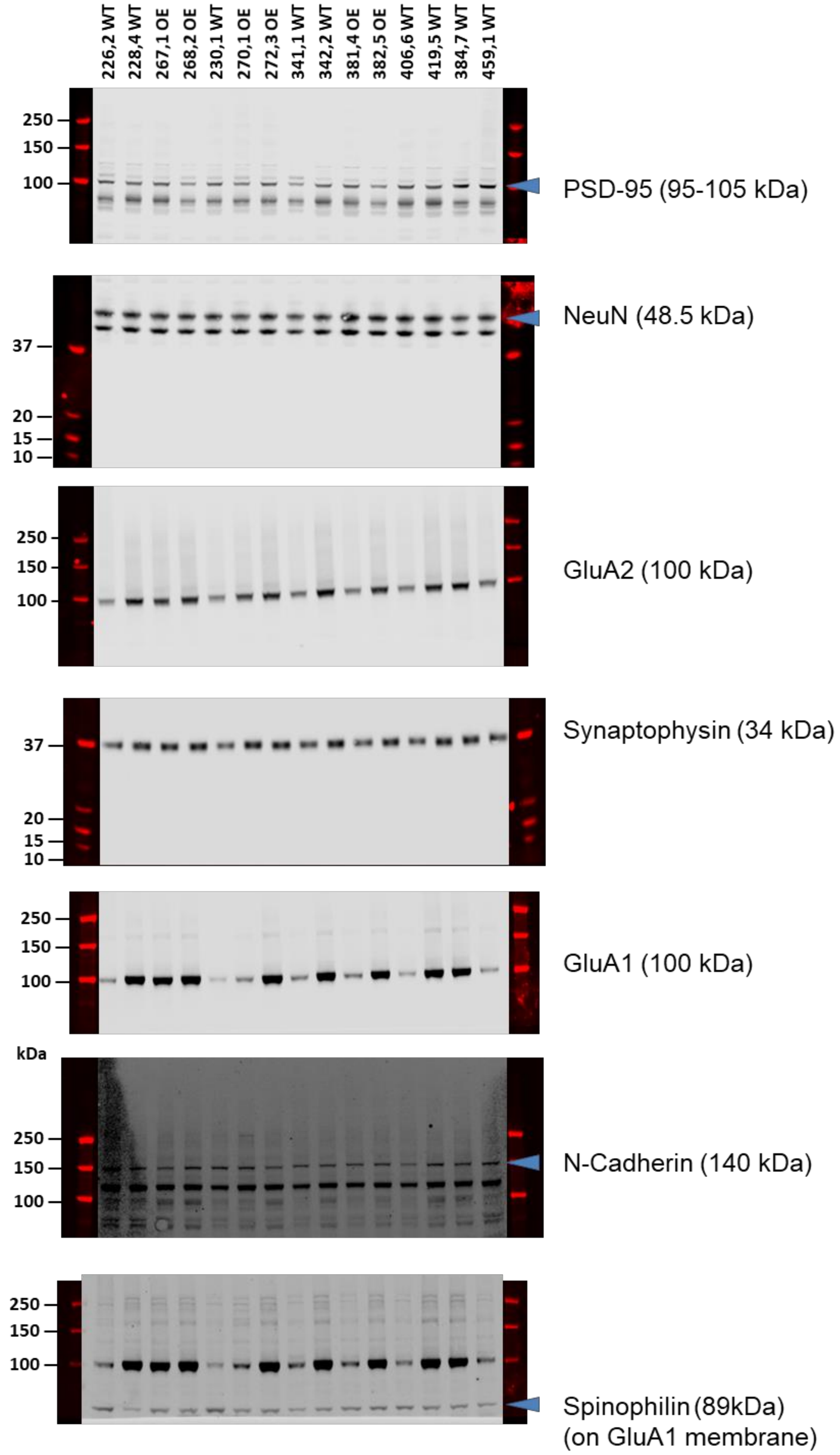

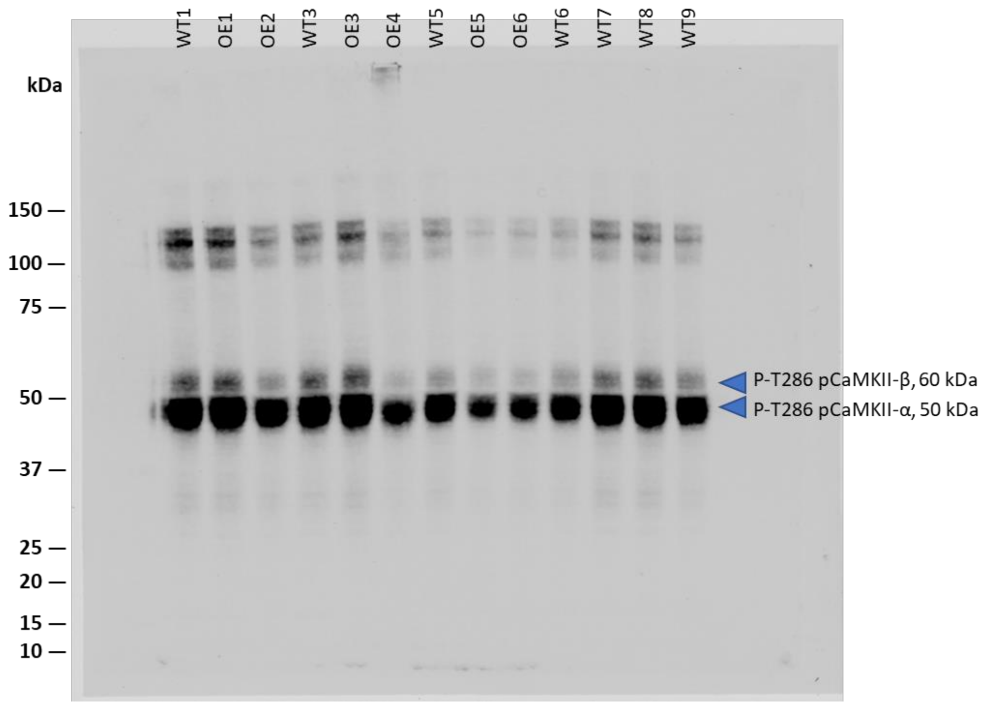
WT1 OE1 OE2 WT3 OE3 OE4 WT5 OE5 OE6 WT6 WT7 WT8 WT9
kDa
150
100
75
50
37
25
20
15
10
P-T286 pCaMKII-β, 60 kDa
P-T286 pCaMKII-α, 50 kDa

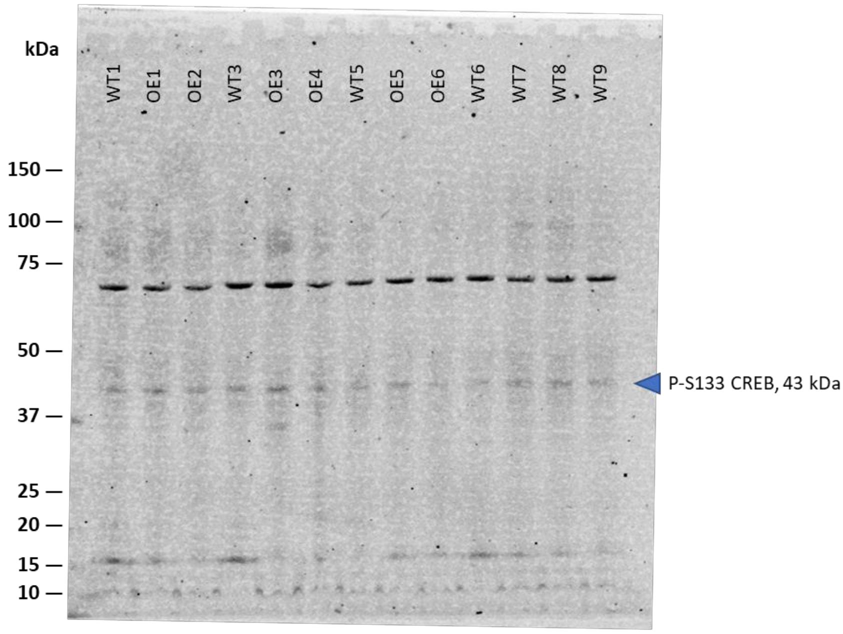
kDa
WT1 OE1 OE2 WT3 OE3 OE4 WT5 OE5 OE6 WT6 WT7 WT8 WT9
150
100
75
50
37
25
20
15
10
P-S133 CREB, 43 kDa

β-Estradiol ELISA assay. Serum β-Estradiol concentration was determined using β-Estradiol ELISA assay. Since the serum had been kept in the freezer for an extended period of time, an exact physiological estradiol concentration cannot be certain and thus the data is presented as relative values. All replicates were measured on the same ELISA plate and the average intra CV is 6.4 %.

**Mouse ID** WT	R1 OD	R2 OD	R1 pg/lm	R2 pg/lm	R1 pg/ml /Ave	R1 pg/ml /Ave	SD	Intra CV %
226	0,94	0,91	12,70	13,78	0,91	0,99	0,055	5,52
228	0,90	0,91	13,96	13,71	1,00	0,98	0,013	1,26
341	0,87	0,89	15,16	14,49	1,09	1,04	0,038	3,80
384	0,92	0,86	13,37	15,31	0,96	1,10	0,034	3,40
342	0,96	0,96	12,26	12,24	0,88	0,88	0,001	0,14
459	0,99	0,92	11,39	13,45	0,82	0,97	0,099	9,88
230	0,92	0,90	13,40	14,15	0,96	1,02	0,060	6,01
406	0,92	0,96	13,44	12,25	0,96	0,88	0,139	13,89
419	0,96	0,87	12,29	15,02	0,88	1,08	0,105	10,47
PA28αOE								
268	0,95	0,89	12,44	14,46	0,89	1,04	0,008	0,81
267	0,90	0,89	14,18	14,34	1,02	1,03	0,102	10,25
382	0,97	0,96	11,78	12,28	0,85	0,88	0,172	17,21
381	0,83	0,81	16,31	17,00	1,17	1,22	0,073	7,33
270	0,90	0,79	14,14	17,54	1,02	1,26	0,035	3,47
272	0,88	0,83	14,84	16,29	1,07	1,17	0,025	2,51
