# Supplementary material for: PA28αβ overexpression enhances learning and memory of female mice without inducing 20S proteasome activity
Source: BMC Neurosci. 2018 Nov 6;19:70. doi: 10.1186/s12868-018-0468-2 (PMC6218978; doi:10.1186/s12868-018-0468-2)
Supplement: Supplementary file 11 — Additional file 11. PA28-dependent proteasome activity of PA28αOE and WT MEFs. [file 12868_2018_468_MOESM11_ESM.pdf]

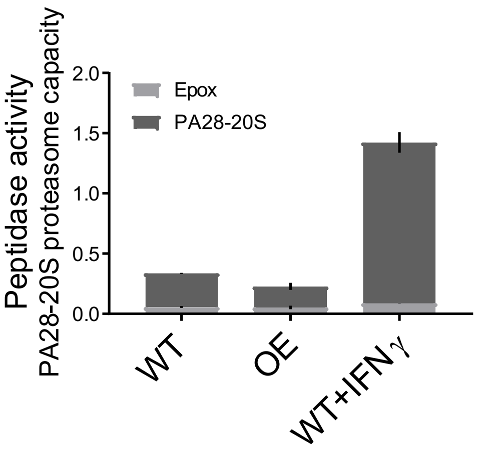

Additional file 11: PA28α overexpression in MEFs does not increase PA28-dependent proteasome activity. PA28-dependent proteasome capacity in PA28αOE and WT MEFs and WT IFN-γ treated MEFs, values are mean±SEM (n=3). Data is presented in full in Additional file 12.
