# Supplementary material for: PA28αβ overexpression enhances learning and memory of female mice without inducing 20S proteasome activity
Source: BMC Neurosci. 2018 Nov 6;19:70. doi: 10.1186/s12868-018-0468-2 (PMC6218978; doi:10.1186/s12868-018-0468-2)
Supplement: Supplementary file 13 — Additional file 13. The raw data used to produce Fig. 4b, 4d, 4e and Additional file 9. [file 12868_2018_468_MOESM13_ESM.pdf]

Sample	*repl 1*	*repl 2*	*repl 3*	*repl 4*	*repl 5*	*repl 6*	*repl 7*	*repl 8*	*repl 9*	mean	m-b	N* EB	x100	inhibited agg
**WT hyb:1**	0,105	0,107	0,099	0,106						0,1042	0,0213	0,982	98,2	1,8
**OE hyb:4**	0,100	0,102	0,100	0,100	0,103					0,1009	0,0180	0,830	83,0	17,0
**WT hyb:7**	0,101	0,107	0,098	0,106	0,101					0,1024	0,0195	0,899	89,9	10,1
**OE hyb:6**	0,101	0,102	0,102	0,100	0,100					0,1009	0,0180	0,829	82,9	17,1
**WT hyb:6**	0,100	0,101	0,101	0,104	0,099					0,1008	0,0179	0,823	82,3	17,7
**OE hyb:5**	0,100	0,100	0,102	0,100	0,096					0,0996	0,0167	0,771	77,1	22,9
**WT hyb:9**	0,102	0,105	0,101	0,100	0,097					0,1009	0,0180	0,829	82,9	17,1
**EB**	0,106	0,104	0,104							0,1046	0,0217	1	100	0
**No luc EB**	0,083	0,083								0,0829	0	0	0	100
**boiled extr.**	0,114	0,111	0,110	0,111	0,112	0,109	0,116			0,1120	0,0291	1,340	134,0	-34,0
**boiled EB**	0,106	0,109								0,1071	0,0242	1,115	111,5	-11,5
**WT C57:1**	0,126	0,120	0,120	0,118	0,116	0,119	0,117	0,117		0,1191	0,0490	0,796	79,6	20,4
**OE C57:1**	0,126	0,118	0,115	0,115	0,112	0,116	0,116	0,119		0,1172	0,0470	0,764	76,4	23,6
**WT C57:2**	0,116	0,121	0,110	0,112	0,118	0,117	0,116	0,118		0,1160	0,0459	0,746	74,6	25,4
**OE C57:2**	0,117	0,119	0,115	0,108	0,112	0,116	0,118	0,120		0,1158	0,0456	0,742	74,2	25,8
**WT C57:3**	0,119	0,117	0,114	0,107	0,117	0,115	0,113	0,116		0,1149	0,0447	0,727	72,7	27,3
**OE C57:3**	0,114	0,112	0,112	0,114	0,113	0,120	0,117	0,116		0,1146	0,0445	0,723	72,3	27,7
**WT C57:4**	0,107	0,108	0,105	0,103	0,107	0,106	0,109	0,113		0,1070	0,0368	0,598	59,8	40,2*
**OE C57:4**	0,120	0,112	0,107	0,114	0,113	0,112	0,114	0,109		0,1126	0,0425	0,690	69,0	31,0
**EB**	0,132	0,129	0,131	0,133	0,137	0,131	0,128	0,131		0,1317	0,0615	1	100	0
**no agg**	0,071	0,070	0,069	0,070	0,070	0,070	0,071	0,072	0,068	0,0701	0		0	100
**WT C57:5**	0,117	0,112	0,108	0,110	0,110	0,111	0,113	0,110		0,1113	0,0399	0,844	84,4	15,6
**OE C57:5**	0,109	0,107	0,106	0,105	0,107	0,108	0,109	0,106		0,1072	0,0358	0,759	75,9	24,1
**WT C57:6**	0,111	0,107	0,106	0,102	0,108	0,107	0,108	0,109		0,1074	0,0360	0,762	76,2	23,8
**OE C57:6**	0,108	0,106	0,105	0,106	0,106	0,105	0,108	0,107		0,1064	0,0350	0,741	74,1	25,9
**WT C57:7**	0,110	0,111	0,112	0,112	0,112	0,115	0,114	0,114		0,1127	0,0413	0,874	87,4	12,6
**EB**	0,118	0,115	0,117	0,120	0,124					0,1186	0,0472	1	100	0
**no agg**	0,072	0,072	0,071	0,071	0,072	0,069				0,0714	0		0	100

*This value differs by twice the standard deviation or more from the mean, the only value to do so, and was therefore identified as an outlier and excluded from the analysis.

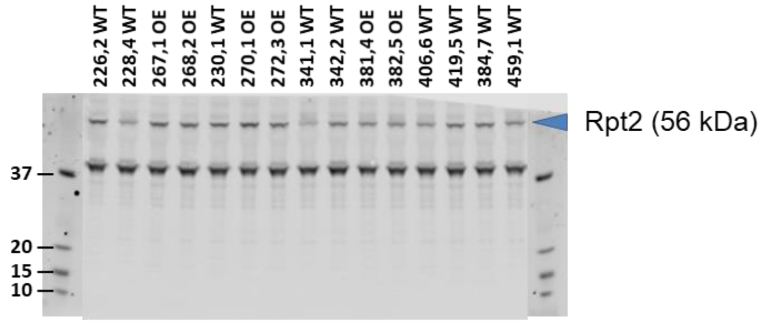

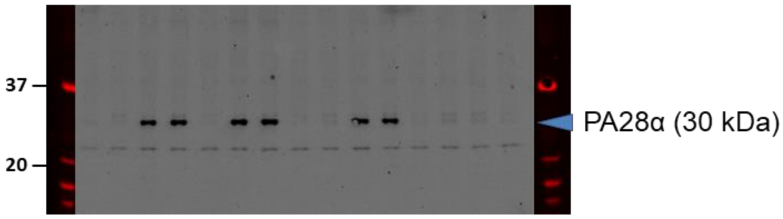

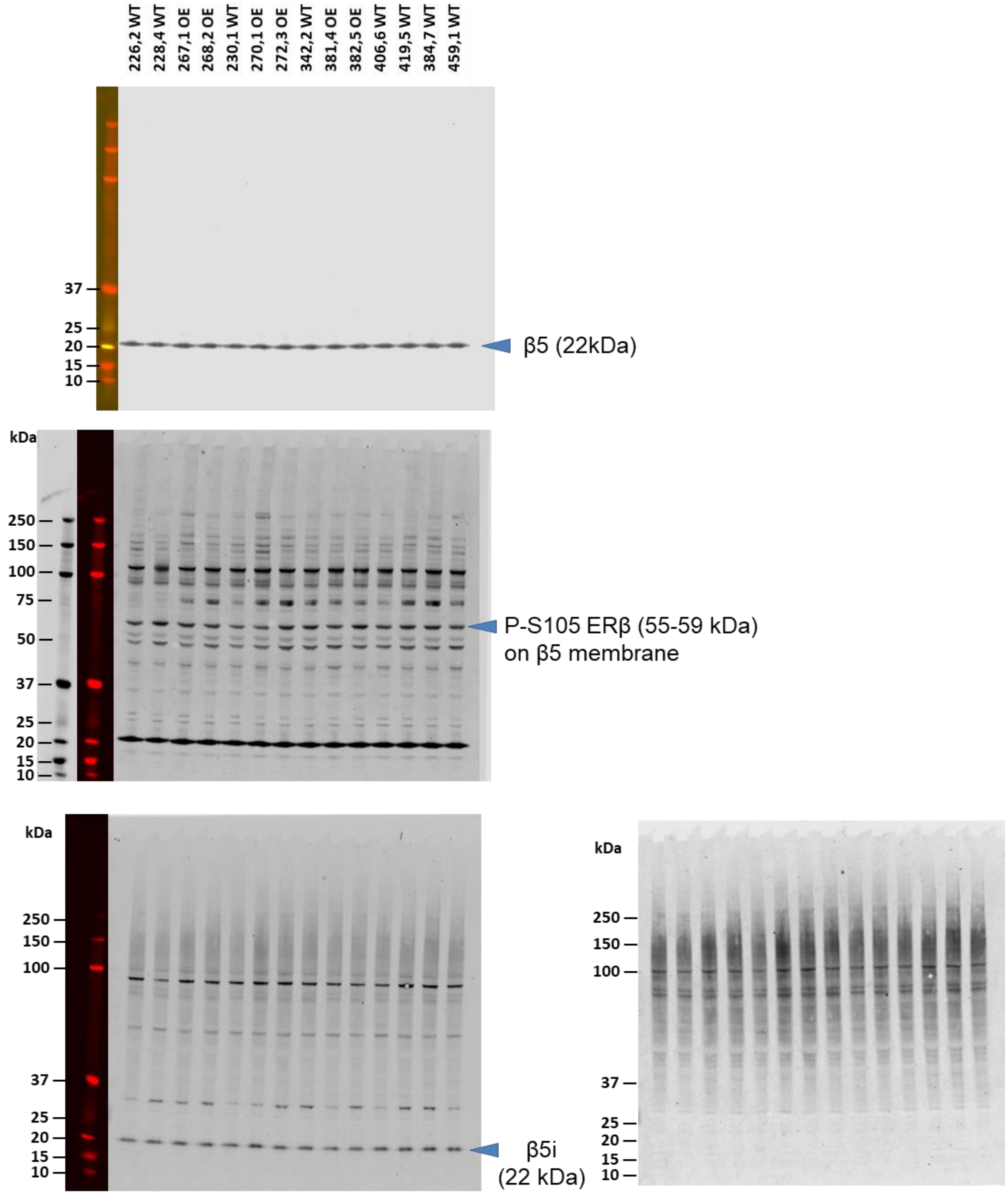

Additional file 13: Raw data to figure 4 and Additional file 9. Upper 1st page: Raw data table for aggregation prevention shown in Fig. 4e). “hyb” = C57BL/6N×BALB/c F2 hybrids; “C57” = C57BL/6N mice; “luc” = heat sensitive luciferase; “EB” = extraction buffer; “no agg” = value prior 42°C; “m-b” = mean value subtracted by background value (No luc EB or no agg); “N* EB” = normalized to 100% aggregation value (EB). Lower 1st page: Full-length blots of cropped blots of Rpt2 and PA28α shown in Fig. 4d. Blots were cut in half prior immuno-detection. This page: Full-length blots of cropped blots of β5, P-S105 ERβ and β5 shown in Fig. 4d and Additional file 9. Due to limited amount of protein extract, the membrane used for detection of K48-polyubiquitination was reused to detect β5i; K48-polyubiquitination membrane is included to the right for reference. Identification of band corresponding to protein of interest was based on molecular weight and remaining bands were considered background detection.
