# Supplementary material for: PA28αβ overexpression enhances learning and memory of female mice without inducing 20S proteasome activity
Source: BMC Neurosci. 2018 Nov 6;19:70. doi: 10.1186/s12868-018-0468-2 (PMC6218978; doi:10.1186/s12868-018-0468-2)
Supplement: Supplementary file 14 — Additional file 14. Sequences of primers used for real-time quantitative (qPCR) analysis. [file 12868_2018_468_MOESM14_ESM.pdf]

Additional file 14: Table S2

Sequences of primers used for real-time quantitative (qPCR) analysis.

Detected mRNA	Sequence
PA28α	Forward Primer: 5´-GTACCCGATCCAGTCAAAGAGA-3´<br>Reverse Primer: 5´-CTCAGGTTTTAGGCGTTGCAG-3´
PA28β v1	Forward Primer: 5´-GAGAAGCCCGAAAACAGGTG-3´<br>Reverse Primer: 5´-AGAGCTGACTCAGGGATATGATT-3´
PA28β v1	Forward Primer: 5´-CACTTTCTTGCCACGGAAAATC-3´<br>Reverse Primer: 5´-GAGGATCTGGGATAGGGATGTC-3´
36b4	Forward Primer: 5´-CGA CCT GGA AGT CCA ACT AC-3´<br>Reverse Primer: 5´-ATC TGC TGC ATC TGC TTG-3´

All primer sequences except 36b4 were obtained from the PrimerBank database[1-4].

REFERENCES TO ADDITIONAL FILE TABLE S2

1 Wang, X., Spandidos, A., Wang, H. & Seed, B. PrimerBank: a PCR primer database for quantitative gene expression analysis, 2012 update. *Nucleic Acids Research* **40**, D1144-D1149, doi:10.1093/nar/gkr1013 (2012).

2 Spandidos, A., Wang, X., Wang, H. & Seed, B. PrimerBank: a resource of human and mouse PCR primer pairs for gene expression detection and quantification. *Nucleic Acids Research* **38**, D792-D799, doi:10.1093/nar/gkp1005 (2010).

3 Spandidos, A. *et al.* A comprehensive collection of experimentally validated primers for Polymerase Chain Reaction quantitation of murine transcript abundance. *BMC Genomics* **9**, 633-633, doi:10.1186/1471-2164-9-633 (2008).

4 Wang, X. & Seed, B. A PCR primer bank for quantitative gene expression analysis. *Nucleic Acids Research* **31**, e154-e154, doi:10.1093/nar/gng154 (2003).
